# Genetic differentiation of Pisang Awak subvarieties and genetic variation among ‘Mali-Ong’ plantlets in Thailand using RAPD and SRAP markers

**DOI:** 10.1016/j.jgeb.2025.100577

**Published:** 2025-10-03

**Authors:** Thanita Boonsrangsom, Kawee Sujipuli, Duangporn Premjet

**Affiliations:** a Department of Agricultural Science, Faculty of Agriculture, Natural Resources and Environment, Naresuan University, Phitsanulok 65000, Thailand; b Center of Excellence in Research for Agricultural Biotechnology, Naresuan University, Phitsanulok 65000, Thailand

**Keywords:** Pisang Awak, Random amplification of polymorphic DNA (RAPD), Sequence-related amplified polymorphism (SRAP), Clonal variation, Banana germplasm, Genetic diversity, ABB genome group

## Abstract

Banana (*Musa* spp.) is a globally important fruit crop, with most cultivated varieties originating from hybridizations between *M. acuminata* (A genome) and *M. balbisiana* (B genome). Triploid ABB hybrids, carrying two B-genome and one A-genome sets, are valued for their stress tolerance and adaptability. In Thailand, ‘Kluai Namwa’ (Pisang Awak) is the most widely cultivated ABB cultivar, but it shows considerable phenotypic variation across subvarieties. Because morphological classification is often unreliable, molecular tools are needed to assess genetic identity and diversity. A total of 28 Thai banana genotypes, representing the AA, BB, and ABB genome groups, were analyzed using RAPD and SRAP markers. RAPD produced 109 bands with 93.6 % polymorphism, while SRAP generated 278 bands with 92.5 % polymorphism, indicating substantial genetic variation. The mean polymorphic information content was 0.22 for RAPD and 0.24 for SRAP, confirming the discriminatory power of both marker systems. UPGMA clustering separated the genotypes into two major clusters corresponding to A- and B-genome contributions, a structure further supported by PCoA. Distinctive bands, such as RAPD primer S7 (2.50 kb) and SRAP combination Me6/Em8 (0.80 kb), specifically identified ‘Kluai Hak Muk’ cooking bananas, demonstrating the potential of these markers for cultivar authentication. The genetic stability of 16 ‘Kluai Namwa Mali-Ong’ plantlets from different locations was also evaluated. Results revealed high clonal uniformity (mean similarity = 0.858) with only minor variation, likely reflecting localized cultivation practices. Overall, RAPD and SRAP markers proved effective for genome identification, diversity assessment, and clonal stability monitoring in Thai bananas. These tools will support banana breeding, germplasm conservation, and reliable authentication of high-value cultivars.

## Highlights

• RAPD and SRAP markers revealed >92 % polymorphism among 28 Thai *Musa* genotypes.
• Moderate PIC values confirmed substantial genetic variation across AA, BB, and ABB groups.
• UPGMA and PCoA consistently grouped genotypes according to A- and B-genome contributions.
• Unique RAPD (S7) and SRAP (Me6/Em8) bands identified the cooking banana ‘Kluai Hak Muk’ (Bluggoe).
• ‘Kluai Namwa Mali-Ong’ plantlets showed high clonal uniformity (mean similarity = 0.858).

## Graphical abstract

**Figure:**
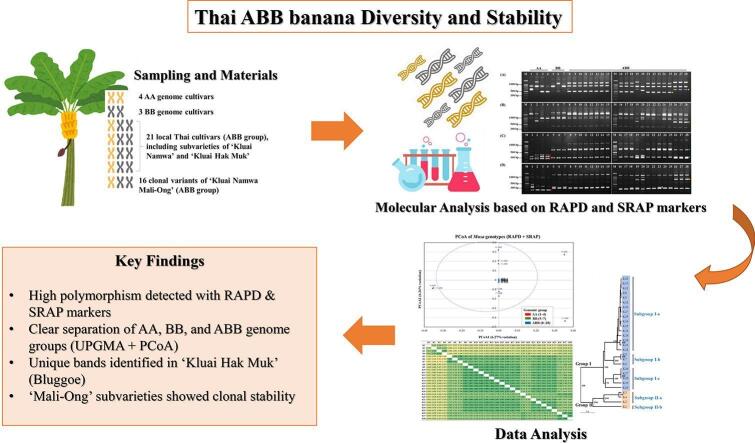

## 1 Introduction

Bananas (*Musa* spp.) are one of the most important fruit crops globally, playing a vital role in supporting rural livelihoods, nutrition, and food security, particularly in tropical and subtropical regions.^1^ In Thailand, bananas are a staple food and a major agricultural commodity with cultural and economic significance. Smallholder farmers significantly contribute to the annual production of over 1.3 million tons, with ‘Kluai Namwa’ being a primary cultivar.^2^ Besides local consumption, bananas are processed into value-added products, such as solar-dried bananas, banana chips, and banana flour, supporting rural economies and the agro-industrial sector.

Among the diverse banana cultivars, triploid hybrids of the ABB genomic group, which comprise two chromosome sets from *Musa balbisiana* (BB) and one from *M. acuminata* (A), are notable for their resilience, including stress tolerance, disease resistance, and environmental adaptability. One of the most important ABB cultivars in Southeast Asia is Pisang Awak, known in Thailand as ‘Kluai Namwa’. This traditional cultivar likely originated from natural hybridization between *M. acuminata* and *M. balbisiana*, with the latter serving as the female parent. A non-disjunction event during fertilization led to its triploid chromosome number (2n = 3x = 33). Because of its adaptability and drought tolerance, ‘Kluai Namwa’ has long been cultivated in Thailand, where it is believed to have originated.^3^ The Namwa banana exhibits a remarkable degree of genetic variation, with more than ten subvarieties differing slightly in traits such as peel color, flesh color, plant height, and overall morphology. However, differentiation of these subvarieties is a challenge, as these variations can be subtle and influenced by environmental conditions. One notable subvariety, ‘Kluai Namwa Mali-Ong’, is highly valued for its delicate texture and mild sweetness. It is not only popular in local markets but also widely used in community-based products in Phitsanulok Province, contributing significantly to local economies. However, the growing demand for this subvariety often exceeds local production capacity, leading to imports from neighboring countries that may compromise product quality, particularly in processed forms.

Genetic diversity is essential for crop improvement, conservation, and resilience to environmental stress and diseases.^4^, ^5^, ^6^ In bananas, this diversity arises from hybridization between *M. acuminata* and *M. balbisiana* progenitors, resulting in different genomic groups (AAA, ABB, AAB) and ploidy levels (diploid, triploid, tetraploid).^7^ Southeast Asia, a primary center of banana diversity, holds numerous locally cultivated varieties that remain insufficiently characterized, including ‘Kluai Namwa Mali-Ong’.^8^, ^9^ Understanding genetic differentiation and stability is crucial for conservation and breeding programs, particularly despite the long cultivation history and regional planting material exchanges in Thailand.

Traditional classification methods, such as morphological analysis, are often limited in resolution due to the influence of environmental conditions and cultivation practices, highlighting the need for more accurate and robust molecular approaches. Numerous studies have examined genetic variation across multiple *Musa* species using a variety of molecular markers.^10^, ^11^, ^12^, ^13^, ^14^ Molecular markers provide reliable tools for characterizing genetic diversity, population structure, and stability. Among these, random amplification of polymorphic DNA (RAPD) and sequence-related amplified polymorphism (SRAP) markers are widely used due to their complementary advantages.^15^, ^16^, ^17^ RAPD is a rapid and cost-effective technique that generates random DNA fragments using short primers,^18^, ^19^ though its reproducibility can be influenced by experimental conditions.^20^, ^21^ In contrast, SRAP markers target coding regions and amplify open reading frames, offering insights into functional genetic variation related to traits like stress tolerance and agronomic performance.^22^, ^23^, ^24^

Local triploid bananas (ABB group) play a crucial role in Thailand’s agriculture, particularly as staple crops and income sources for smallholder farmers. However, genetic differentiation and stability within these cultivars are still poorly understood. Due to the increasing demand for high-quality banana cultivars in both local and international markets, a comprehensive genetic assessment of ABB bananas in Thailand is necessary. This study aims to assess the genetic differentiation among Pisang Awak (ABB group) subvarieties and evaluate the genetic variation among ‘Kluai Namwa Mali-Ong’ plantlets collected from different locations in Thailand. Using RAPD and SRAP molecular markers, the research provides insights into genetic diversity and clonal variation, contributing to the conservation and sustainable use of banana germplasm under changing agricultural and environmental conditions.

## 2 Material and methods

### 2.1 Plant materials

A total of 43 Thai *Musa* genotypes, representing three genomic groups (AA, BB, and ABB), were analyzed in this study (Table 1). These comprised 28 local cultivars (samples 1–28) selected to represent the major banana types cultivated in Thailand, and 15 samples of the ABB subvariety ‘Kluai Namwa Mali-Ong’ (samples 29–43) included for clonal stability assessment. The AA and BB genotypes were used as parental references and outgroups to anchor comparisons with ABB hybrid triploids.

**Table 1:** List of 43 *Musa* germplasms analyzed in this study.

**No.**	**ID**	**Cultivar name**	**Banana type**	**Genomic group^a^**	**Location^b^**
	***Musa acuminata* (AA group)**				
1	K1	‘Kluai Pa-Phrae’	Dessert	AA	PRSKU
2	K2	‘Kluai Hom Champa’	Dessert	AA	PRSKU
3	K3	‘Kluai Khai Kasetsart 2’	Dessert	AA	PRSKU
4	K4	‘Kluai Khai Khamphaeng Phet’	Dessert	AA	PRSKU

	***Musa balbisiana* (BB group)**				
5	K5	‘Kluai Tani Isan’	Cooking	BB	PRSKU
6	K6	‘Kluai Tani Nuea’	Cooking	BB	PRSKU
7	K7	‘Kluai Tani Dam’	Cooking	BB	PRSKU

	***Musa* × *paradisiaca* (ABB group)**				
8	K8	‘Kluai Namwa Dam’	Dessert/Cooking	ABB	PPC
9	K9	‘Kluai Namwa Ubon’	Dessert/Cooking	ABB	PPC
10	K10	‘Kluai Namwa Ngern’	Dessert/Cooking	ABB	PPC
11	K11	‘Kluai Namwa Mali-Ong’ (ML1)	Dessert/Cooking	ABB	PPC
12	K12	‘Kluai Namwa Pakchong 50’	Dessert/Cooking	ABB	PPC
13	K13	‘Kluai Namwa Khom’	Dessert/Cooking	ABB	PPC
14	K14	‘Kluai Namwa Phrarachthan’	Dessert/Cooking	ABB	PPC
15	K15	‘Kluai Namwa Sai Lueang’	Dessert/Cooking	ABB	PPC
16	K16	‘Kluai Namwa Nuan’	Dessert/Cooking	ABB	PPC
17	K17	‘Kluai Namwa Sai Dam’	Dessert/Cooking	ABB	PPC
18	K18	‘Kluai Namwa Khieo’	Dessert/Cooking	ABB	PPC
19	K19	‘Kluai Nom Mi’	Cooking	ABB	PPC
20	K20	‘Kluai Hin’	Cooking	ABB	PPC
21	K21	‘Kluai Namwa Tha Yang’	Dessert/Cooking	ABB	PPC
22	K22	‘Kluai Namwa Kab Khao’	Dessert/Cooking	ABB	PPC
23	K23	‘Kluai Namwa Suan’	Dessert/Cooking	ABB	PRSKU
24	K24	‘Kluai Nam Wo’	Dessert/Cooking	ABB	PRSKU
25	K25	‘Kluai Namwa Tanao Sri’	Dessert/Cooking	ABB	PRSKU
26	K26	‘Kluai Hak Muk Nuan’	Cooking	ABB	PPC
27	K27	‘Kluai Hak Muk Khieo’	Cooking	ABB	PPC
28	K28	‘Kluai Hak Muk Thong’	Cooking	ABB	PPC
29	ML2	‘Kluai Namwa Mali-Ong’	Dessert/Cooking	ABB	PPC
30	ML3	‘Kluai Namwa Mali-Ong’	Dessert/Cooking	ABB	PPC
31	ML4	‘Kluai Namwa Mali-Ong’	Dessert/Cooking	ABB	PPC
32	ML5	‘Kluai Namwa Mali-Ong’	Dessert/Cooking	ABB	PPC
33	ML6	‘Kluai Namwa Mali-Ong’	Dessert/Cooking	ABB	PPC
34	ML7	‘Kluai Namwa Mali-Ong’	Dessert/Cooking	ABB	PPC
35	ML8	‘Kluai Namwa Mali-Ong’	Dessert/Cooking	ABB	SKM
36	ML9	‘Kluai Namwa Mali-Ong’	Dessert/Cooking	ABB	PBI
37	ML10	‘Kluai Namwa Mali-Ong’	Dessert/Cooking	ABB	SPB
38	ML11	‘Kluai Namwa Mali-Ong’	Dessert/Cooking	ABB	SPB
39	ML12	‘Kluai Namwa Mali-Ong’	Dessert/Cooking	ABB	CBI
40	ML13	‘Kluai Namwa Mali-Ong’	Dessert/Cooking	ABB	PNB
41	ML14	‘Kluai Namwa Mali-Ong’	Dessert/Cooking	ABB	SRI
42	ML15	‘Kluai Namwa Mali-Ong’	Dessert/Cooking	ABB	PTE
43	ML16	‘Kluai Namwa Mali-Ong’	Dessert/Cooking	ABB	PRSKU

**Note:**^a^ Classification of banana genomes based on Silayoi^25^; ^b^ Location of sample collection: PPC – Plant Propagation Center No. 6, Phitsanulok Province; PRSKU – Pakchong Research Station, Kasetsart University, Nakhon Ratchasima Province; SKM – Samut Songkhram Province; PBI – Phetchaburi Province; SPB – Suphan Buri Province; CBI – Chon Buri Province; PNB – Phetchabun Province; SRI – Saraburi Province; PTE – Pathum Thani Province.

Young, healthy leaf samples were collected from two major germplasm conservation sites: (1) the Plant Propagation Center No. 6 (PPC) in Phitsanulok Province, and (2) the Pakchong Research Station of Kasetsart University (PRSKU) in Nakhon Ratchasima Province. To assess genetic stability within ‘Kluai Namwa Mali-Ong,’ additional samples were obtained from different production areas across Thailand, including tissue culture-derived plants (samples 29–34) maintained at the PPC tissue culture laboratory. This sampling strategy ensured broad representation of genome-level diversity (AA, BB, and ABB) while enabling specific evaluation of clonal uniformity within the high-value ‘Kluai Namwa Mali-Ong’ subvariety.

### 2.2 Genomic DNA isolation and quality assessment

Genomic DNA was extracted from fresh young banana leaves using the BioFACT™ Genomic DNA Prep Kit for Plant (BIOFACT Co., Ltd., Korea) following the manufacturer’s protocol with minor modifications. Approximately 100 mg of leaf tissue was ground to a fine powder in liquid nitrogen using a pre-chilled mortar and pestle. The powder was mixed with 0.5 mL of lysis buffer supplemented with 5 µL of Proteinase K (20 mg/mL) and 2 µL of RNase A (4 mg/mL), followed by incubation at 65 °C for 30 min. DNA was precipitated with cooled isopropanol and collected by centrifugation at 14,000 × *g* for 30 s. The resulting pellet was washed twice with the supplied washing solution, air-dried, and dissolved in 50 µL of TE buffer.

DNA integrity was verified by electrophoresis on a 1.0 % agarose gel stained with Safe DNA Dye (Hydragreen™, USA). DNA purity and concentration were assessed spectrophotometrically using a Synergy H1 Microplate Reader (BioTek, USA), based on absorbance at A_260_ nm (nucleic acid content) and the A_260_/A_280_ ratio (protein contamination). The A_260_/A_280_ ratios of the extracted DNA ranged from 1.80 to 1.90, confirming high purity. Only DNA samples with an A_260_/A_280_ ratio between 1.8 and 2.0 and concentrations above 50 ng/µL were used for PCR amplification. The genomic DNA was stored at –20 °C until further use.

### 2.3 RAPD and SRAP PCR amplification and genotyping

A pre-screening of 40 RAPD primers (S1–S40) was conducted using a Bio-Rad T100™ thermal cycler (USA). The RAPD-PCR reactions were carried out in a total volume of 20 µL, containing 50 ng of template DNA, 10 µL of 2× Quick Taq™ HS DyeMix (Toyobo Co., Ltd., Japan), and 5 µM of a RAPD primer (BioBasic Inc., Canada). The PCR program included an initial denaturation at 94 °C for 3 min, followed by 40 cycles of denaturation at 94 °C for 1 min, annealing at 37 °C for 1 min, and extension at 68 °C for 2 min, with a final extension at 68 °C for 10 min. Primers producing clear, reproducible, and polymorphic bands were selected, resulting in 13 primers used for final analysis. Each RAPD reaction was performed in two independent PCR runs, and electrophoresis was repeated once to confirm reproducibility of banding patterns. Only consistently reproducible bands were scored.

For SRAP analysis, 56 primer combinations, consisting of seven forward primers (Me1–Me7) and eight reverse primers (Em1–Em8),^22^ were tested. The SRAP-PCR reactions were conducted in a total volume of 20 µL, containing 50 ng of template DNA, 2× Quick Taq™ HS DyeMix (Toyobo Co., Ltd., Japan), and 5 µM each of SRAP forward and reverse primers. The amplification protocol started with an initial denaturation at 94 °C for 3 min, followed by 5 cycles of denaturation at 94 °C for 1 min, annealing at 35 °C for 1 min, and extension at 68 °C for 2 min. This was followed by 35 cycles of denaturation at 94 °C for 1 min, annealing at 50 °C for 1 min, and extension at 68 °C for 2 min, with a final extension at 68 °C for 10 min. Of the 56 combinations, 28 that consistently produced clear and polymorphic banding patterns were retained for analysis. Each SRAP reaction was likewise repeated in two independent PCR runs, and electrophoresis was performed in duplicate to confirm reproducibility.

The amplified RAPD and SRAP products were separated on a 1.2 % agarose gel in 1× TAE buffer and stained with Safe DNA Dye (Hydragreen™, USA). Gel images were captured using a gel documentation system (Thermo Fisher Scientific, USA).

### 2.4 Data analysis

The RAPD and SRAP PCR products were scored as binary data based on the presence (1) or absence (0) of distinct and reproducible DNA fragments across all banana samples. Key molecular diversity metrics were calculated, including the total number of amplified fragments (NA), number of polymorphic fragments (NP), percentage of polymorphic bands (PPB%), and the average number of bands per marker. The polymorphism information content (PIC) for each marker was calculated using the formula: PIC = 1 − [f^2^ + (1 − f)^2^], where *f* is the frequency of the marker in the dataset.^26^ For dominant markers, the maximum PIC value is 0.5.

To further evaluate the efficiency and discriminatory power of the marker systems, the Effective Multiplex Ratio (EMR) and Marker Index (MI) were calculated. EMR was determined as the product of the total number of amplified bands (NA) and the proportion of polymorphic bands (β = NP/NA), representing the effective number of polymorphic loci per assay. The Marker Index (MI), which combines both the informativeness and efficiency of a marker system, was calculated by multiplying the average PIC by the EMR (MI = PIC × EMR).^27^ These parameters provide an integrated assessment of each marker system’s effectiveness in revealing genetic variation among *Musa* genotypes.

To assess genetic differentiation among banana genotypes, pairwise genetic similarity was calculated using Nei and Li’s similarity coefficient.^28^ Similarity matrices were generated separately for RAPD, SRAP, and combined datasets. Cluster analysis was performed using the Unweighted Pair Group Method with Arithmetic Mean (UPGMA) in FreeTree software, and dendrograms were visualized with TreeView.^29^ Bootstrap analysis with 1000 replicates was conducted to evaluate the robustness of clustering, with bootstrap values above 70 % considered indicative of strong support.

To further examine genetic relationships, a Principal Coordinates Analysis (PCoA) was performed using the pairwise Nei–Li genetic distance matrix derived from RAPD and SRAP data. The analysis was conducted by classical multidimensional scaling (MDS) of the distance matrix, and eigenvalue decomposition was applied to obtain the principal coordinates. The proportion of total variation explained by each axis was calculated from the eigenvalues. The first two coordinates were used to visualize genetic variation among genotypes, with samples labeled by number (Table 1) and their genomic group designation (AA, BB, or ABB).

## 3 Result

High-quality genomic DNA was successfully extracted from all banana leaf samples. The DNA showed intact, high-molecular-weight bands on 1 % agarose gel (Supplementary Fig. S1). Spectrophotometric analysis indicated that the DNA was of sufficient purity for PCR amplification, with A_260_/A_280_ ratios ranging from 1.78 to 1.85. DNA concentrations varied between 80 and 200 ng/µL, ensuring consistent template availability across all genotypes.

### 3.1 Genetic differentiation of Thai banana genotypes based on RAPD markers

**Fig. 1:**
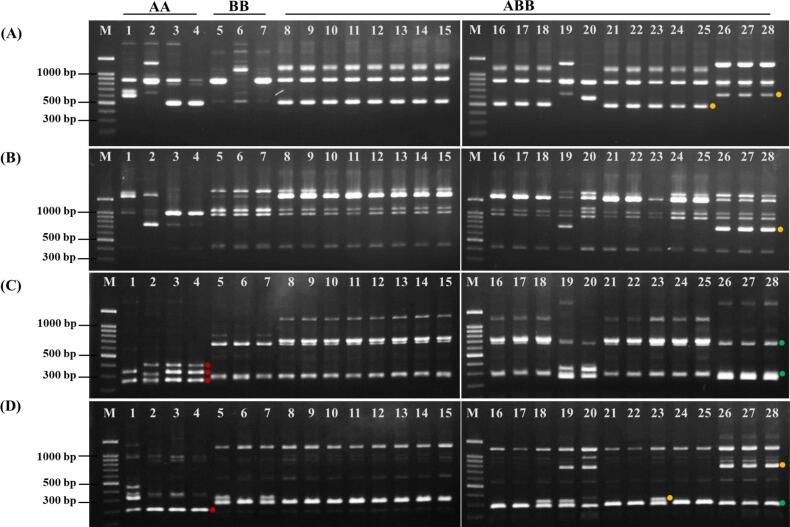
DNA amplification profiles of 28 *Musa* genotypes using RAPD primers S12 (A) and S18 (B) and SRAP primer combinations Me5/Em3 (C) and Me6/Em8 (D). Lane M represents a 100 bp Plus DNA ladder (OneMARK, BIO-HELIX, Taiwan), while Lanes 1–28 correspond to the *Musa* genotypes listed in Table 1. Red dots indicate bands specific to *M. acuminata* (A genome), green dots highlight bands unique to *M. balbisiana* (B genome), and yellow dots mark bands distinguishing triploid hybrid bananas (ABB group) (lanes 8–28).

A total of 40 RAPD primers (S1–S40) were initially screened, of which 13 primers (S1, S6, S7, S8, S11, S12, S17, S18, S25, S28, S31, S33, and S38) (32.5 %) were selected based on their ability to produce clear, distinct, and reproducible DNA bands. The amplified DNA fragments ranged in size from 0.25 to 4.50 kb. Primer S17 generated the highest number of bands (14), with an average of 8.38 bands per primer, while primer S6 produced the fewest (3) (Table 2). Eight primers (S1, S6, S8, S11, S12, S25, S28, and S38) exhibited a 100 % polymorphism rate. Across all 13 selected primers, a total of 109 DNA bands were generated, of which 102 were polymorphic, resulting in a polymorphism rate of 93.58 %. The number of polymorphic bands per primer ranged from 3 (S6 and S31) to 13 (S11), with an average of 7.85. The PIC values varied from 0.11 (S6) to 0.31 (S28), with an average PIC of 0.22. A total EMR of 102.0 and an overall MI of 22.52 were obtained, with average EMR and MI values of 7.85 and 1.73 per primer, respectively. Representative RAPD amplification profiles generated with primers S12 and S18 are shown in Fig. 1A and B.

**Table 2:** Characteristics of RAPD primers used for DNA amplification in 28 *Musa* genotypes, including allele size range, polymorphism percentage, PIC values, effective multiplex ratio (EMR), and marker index (MI).

**No.**	**Primer name**	**Sequence (5**′**–3**′**)**	**Allele size range (kb)**	**NA**	**NP**	**PPB (%)**	**PIC**	**EMR**	**MI**
1	S1	GTTTCGCTCC	0.50–2.30	8	8	100.0	0.19	8	1.52
2	S6	TGCTCTGCCC	0.90–1.80	3	3	100.0	0.11	3	0.33
3	S7	GGTGACGCAG	0.50–2.50	9	8	88.89	0.23	8	1.84
4	S8	GTCCACACGG	0.35–3.00	7	7	100.0	0.22	7	1.54
5	S11	GTAGACCCGT	0.45–4.50	13	13	100.0	0.24	13	3.12
6	S12	CCTTGACGCA	0.50–1.20	9	9	100.0	0.23	9	2.07
7	S17	AGGGAACGAG	0.40–3.00	14	12	85.71	0.24	12	2.88
8	S18	CCACAGCAGT	0.40–3.00	8	7	87.50	0.18	7	1.26
9	S25	AGGGGTCTTG	0.25–1.60	11	11	100.0	0.27	11	2.97
10	S28	GTGACGTAGG	0.35–2.00	9	9	100.0	0.31	9	2.79
11	S31	CAATCGCCGT	0.80–1.50	4	3	75.00	0.21	3	0.63
12	S33	CAGCACCCAC	0.60–1.60	7	5	71.43	0.18	5	0.90
13	S38	AGGTGACCGT	0.35–1.20	7	7	100.0	0.26	7	1.82
	**Total**		**0.25–4.50**	**109**	**102**	**−**	**−**	**102**	**22.52**
	**Average**			**8.38**	**7.85**	**93.58**	**0.22**	**7.85**	**1.73**

**Note:** NA, total number of amplified fragments; NP, total number of polymorphic fragments; PPB, Percentage of polymorphic fragments (%); PIC, Polymorphism information content; EMR, Effective multiplex ratio; MI, Marker index.

### 3.2 Genetic differentiation of Thai banana genotypes based on SRAP markers

A total of 56 SRAP primer combinations, derived from 7 forward and 8 reverse primers, were initially screened using pooled DNA from ‘Kluai Namwa’ genotypes. Based on their consistently high levels of polymorphism and PCR reproducibility, 28 primer combinations were selected for detailed analysis. These selected primers produced a total of 278 amplified fragments, ranging from 0.10 to 3.00 kb in size, with an average of 9.93 fragments per combination (Table 3). Out of the total fragments, 257 were polymorphic, yielding a polymorphism rate of 92.45 %, with an average of 9.18 polymorphic fragments per combination. The primer pair Me4/Em8 produced the highest number of fragments (16), followed by Me1/Em4 and Me2/Em5 (14 each), while Me5/Em1 yielded the fewest (4). Notably, 12 primer combinations exhibited 100 % polymorphism. The PIC values ranged from 0.16 (Me1/Em5 and Me6/Em4) to 0.34 (Me5/Em3), with an average PIC value of 0.24. The total EMR was 257.0, and the overall MI was calculated as 61.50. The average EMR and MI per primer pair were 9.18 and 2.20, respectively. Among individual primers, Me1/Em8 and Me4/Em8 exhibited the highest MI values (4.03 and 4.05, respectively), indicating their strong discriminatory power. Representative SRAP fingerprints obtained using primers Me5/Em3 and Me6/Em8 are presented in Fig. 1C and D.

**Table 3:** Characteristics of SRAP primers used for DNA amplification in 28 *Musa* genotypes, including allele size range, polymorphism percentage, PIC values, effective multiplex ratio (EMR), and marker index (MI).

**No.**	**Primer name**	**Sequence (5**′**–3**′**)**	**Allele size range (kb)**	**NA**	**NP**	**PPB (%)**	**PIC**	**EMR**	**MI**
1	Me1/Em1	Me1: TGAGTCCAAACCGG**ATA** Em1: GACTGCGTACGAATT**AAT**	0.20–1.20	11	11	100.0	0.32	11	3.52
2	Me1/Em2	Me1: TGAGTCCAAACCGG**ATA** Em2: GACTGCGTACGAATT**TGC**	0.20–1.80	9	8	88.89	0.18	8	1.44
3	Me1/Em3	Me1: TGAGTCCAAACCGG**ATA** Em3: GACTGCGTACGAATT**GAC**	0.20–2.00	11	11	100.0	0.27	11	2.97
4	Me1/Em4	Me1: TGAGTCCAAACCGG**ATA** Em4: GACTGCGTACGAATT**TGA**	0.20–3.00	14	13	92.86	0.24	13	3.12
5	Me1/Em5	Me1: TGAGTCCAAACCGG**ATA** Em5: GACTGCGTACGAATT**AAC**	0.15–1.50	10	9	90.00	0.16	9	1.44
6	Me1/Em6	Me1: TGAGTCCAAACCGG**ATA** Em6: GACTGCGTACGAATT**GCA**	0.20–1.00	11	11	100.0	0.26	11	2.86
7	Me1/Em8	Me1: TGAGTCCAAACCGG**ATA** Em8: GACTGCGTACGAATT**CTG**	0.18–2.50	13	13	100.0	0.31	13	4.03
8	Me2/Em1	Me2: TGAGTCCAAACCGG**AGC** Em1: GACTGCGTACGAATT**AAT**	0.20–0.60	6	6	100.0	0.22	6	1.32
9	Me2/Em5	Me2: TGAGTCCAAACCGG**AGC** Em5: GACTGCGTACGAATT**AAC**	0.18–2.30	14	12	85.71	0.25	12	3.00
10	Me2/Em7	Me2: TGAGTCCAAACCGG**AGC** Em7: GACTGCGTACGAATT**CAA**	0.10–0.90	13	11	84.62	0.22	11	2.42
11	Me3/Em1	Me3: TGAGTCCAAACCGG**AAT** Em1: GACTGCGTACGAATT**AAT**	0.20–1.60	10	10	100.0	0.27	10	2.70
12	Me3/Em7	Me3: TGAGTCCAAACCGG**AAT** Em7: GACTGCGTACGAATT**CAA**	0.20–1.20	6	6	100.0	0.26	6	1.56
13	Me3/Em8	Me3: TGAGTCCAAACCGG**AAT** Em8: GACTGCGTACGAATT**CTG**	0.25–1.30	8	8	100.0	0.29	8	2.32
14	Me4/Em2	Me4: TGAGTCCAAACCGG**ACC** Em2: GACTGCGTACGAATT**TGC**	0.15–1.50	8	6	75.00	0.21	6	1.26
15	Me4/Em7	Me4: TGAGTCCAAACCGG**ACC** Em7: GACTGCGTACGAATT**CAA**	0.28–0.60	6	5	83.33	0.22	5	1.10
16	Me4/Em8	Me4: TGAGTCCAAACCGG**ACC** Em8: GACTGCGTACGAATT**CTG**	0.15–1.40	16	15	93.75	0.27	15	4.05
17	Me5/Em1	Me5: TGAGTCCAAACCGG**AAG** Em1: GACTGCGTACGAATT**AAT**	0.30–0.70	4	4	100.0	0.27	4	1.08
18	Me5/Em3	Me5: TGAGTCCAAACCGG**AAG** Em3: GACTGCGTACGAATT**GAC**	0.25–2.00	9	9	100.0	0.34	9	3.06
19	Me5/Em8	Me5: TGAGTCCAAACCGG**AAG** Em8: GACTGCGTACGAATT**CTG**	0.30–1.00	9	7	77.78	0.20	7	1.40
20	Me6/Em2	Me6: TGAGTCCAAACCGG**TAA** Em2: GACTGCGTACGAATT**TGC**	0.20–1.60	11	10	90.91	0.23	10	2.30
21	Me6/Em3	Me6: TGAGTCCAAACCGG**TAA** Em3: GACTGCGTACGAATT**GAC**	0.35–1.40	10	10	100.0	0.20	10	2.00
22	Me6/Em4	Me6: TGAGTCCAAACCGG**TAA** Em4: GACTGCGTACGAATT**TGA**	0.30–1.20	6	5	83.33	0.16	5	0.80
23	Me6/Em5	Me6: TGAGTCCAAACCGG**TAA** Em5: GACTGCGTACGAATT**AAC**	0.20–1.50	10	9	90.00	0.24	9	2.16
24	Me6/Em7	Me6: TGAGTCCAAACCGG**TAA** Em7: GACTGCGTACGAATT**CAA**	0.19–1.50	10	9	90.00	0.23	9	2.07
25	Me6/Em8	Me6: TGAGTCCAAACCGG**TAA** Em8: GACTGCGTACGAATT**CTG**	0.20–1.20	10	9	90.00	0.24	9	2.16
26	Me7/Em1	Me7: TGAGTCCAAACCGG**TCC** Em1: GACTGCGTACGAATT**AAT**	0.20–1.60	11	11	100.0	0.24	11	2.64
27	Me7/Em2	Me7: TGAGTCCAAACCGG**TCC** Em2: GACTGCGTACGAATT**TGC**	0.15–1.20	10	9	90.00	0.20	9	1.80
28	Me7/Em4	Me7: TGAGTCCAAACCGG**TCC** Em4: GACTGCGTACGAATT**TGA**	0.10–1.50	12	10	83.33	0.20	10	2.00
	**Total**		**0.10–3.00**	**278**	**257**	**−**	**−**	**257**	**61.50**
	**Average**			**9.93**	**9.18**	**92.45**	**0.24**	**9.18**	**2.20**

**Note:** NA, total number of amplified fragments; NP, total number of polymorphic fragments; PPB, Percentage of polymorphic fragments (%); PIC, Polymorphism information content; EMR, Effective multiplex ratio; MI, Marker index.

### 3.3 Identification of genome-specific DNA markers

Several genome-specific DNA bands were identified among the 28 *Musa* genotypes using both RAPD and SRAP markers (Table 4). These bands provided insights into the differentiation and inheritance patterns of the A and B genomes in diploid and triploid banana genotypes. Among the RAPD primers, S1 produced a 0.50 kb band consistently present in all BB and ABB genotypes but absent in AA diploids, while S6 generated a 0.90 kb band showing a similar distribution. RAPD S7 amplified a 2.50 kb band exclusively in the cooking banana genotypes (K19, K20, K26–K28), among which K26–K28 are classified as ‘Kluai Hak Muk’ or ‘Bluggoe’. RAPD S12 revealed multiple genome-associated bands. For example, a 1.20 kb band was detected in ‘Kluai Nom Mi’ (K19), ‘Kluai Hak Muk’ (K26–K28) and one AA diploid (K2), whereas a 0.50 kb band was found in most ‘Kluai Namwa’ or ‘Pisang Awak’ (ABB) genotypes but was absent in the cooking banana types.

**Table 4:** Identification of genome-specific bands in *Musa* genotypes using RAPD and SRAP markers.

**No.**	**Marker type**	**Primer**	**DNA band size (kb)**	**Specific to genome/genotype**	**Genotype(s)**	**Remarks**
1	RAPD	S1	0.50	B genome	BB, ABB	Present in all BB and ABB genotypes; absent in AA diploids
2		S6	1.80	Predominantly A genome	Most AA genotypes	Absent in K1; partially specific to A genome
3		S6	0.90	B genome	BB, ABB	Present in all BB and ABB genotypes; absent in AA diploids
4		S7	2.50	B genome	K19, K20, K26–K28	Specific to ‘Bluggoe’ ABB genotypes
5		S12	1.20	A genome	K2, K19, K26–K28	Detected in ‘Bluggoe’ ABB genotypes and one AA diploid (K2)
6		S12	0.65	A genome	K1, K19, K26–K28	Present in AA (K1) and ‘Bluggoe’ ABB group
7		S12	0.50	A genome	Most ABB genotypes (Pisang Awak)	Absent in ‘Bluggoe’ ABB genotypes
8		S18	0.85	A genome	K2–K4, K19, K26–K28	Shared between AA diploids and several ABB genotypes
9	SRAP	Me1/Em1	1.00	A genome	AA, ABB	Detected in all AA diploids and several ABB genotypes
10		Me1/Em1	0.75	B genome	BB, ABB	Present in BB and several ABB genotypes
11		Me1/Em1	0.20	A genome	K1-K4, K19, K20, K26–K28	Detected in all AA diploids and ‘Bluggoe’ ABB genotypes
12		Me1/Em4	0.80	B genome	K5–K7, K18–K19, K23, K26–K28	Present in BB and several ABB genotypes (‘Bluggoe’)
13		Me1/Em6	0.80	B genome	K5–K11, K13–K14, K16–K19, K21–K28	Polymorphic band found in BB and ABB genotypes
14		Me1/Em6	0.40	B genome	K5, K7–K18, K20–K25	Present in BB and multiple ABB genotypes
15		Me1/Em6	0.35	B genome	K6, K20	Rare band detected only in 1 BB and 1 ABB genotype
16		Me2/Em5	0.60	A genome	K2, K8–K18, K21–K25	Detected in one AA diploid and ‘Pisang Awak’ ABB genotypes
17		Me2/Em5	0.40	A genome	K1, K3–K4, K8–K18, K20–K25	Present in most AA diploids and ‘Pisang Awak’ ABB genotypes
18		Me2/Em5	0.20	A genome	K2, K19, K26–K28	Detected in 'Bluggoe' ABB genotypes and one AA diploid (K2)
19		Me5/Em3	0.75	–	K8–K18, K21–K25	Band present in most ‘Pisang Awak’ ABB genotypes
20		Me5/Em3	0.70	B genome	K5–K28	Widely detected across BB and ABB genotypes
21		Me5/Em3	0.40	A genome	K2–K4	Detected specifically in AA diploids
22		Me5/Em3	0.32	–	K1, K3–K4	present in most AA diploids
23		Me6/Em8	0.80	–	K19–K20, K26–K28	Detected in all ‘Bluggoe’ cooking bananas (ABB)
24		Me6/Em8	0.30	–	K1, K5, K7, K18–K19, K23	Polymorphic band detected across AA and BB genotypes
25		Me6/Em8	0.25	B genome	BB, ABB	Detected in all BB diploids and most ABB genotypes

**Note:** AA diploids = K1–K4, BB diploids = K5–K7, ABB triploids = K8–K28.

SRAP markers also revealed distinct genome-specific patterns among the *Musa* genotypes. The primer pair Me1/Em1 amplified a 1.00 kb band consistently in all AA diploids and several ABB genotypes, and a 0.75 kb band in BB diploids and multiple ABB genotypes. Additionally, a 0.20 kb band from this primer was detected in all AA diploids and all cooking bananas. The Me1/Em4 primer generated a 0.80 kb band specific to BB diploids and several ABB genotypes. Me1/Em6 revealed several B genome-related bands: a 0.80 kb band was polymorphic but present in most BB and ABB genotypes, a 0.40 kb band was found across a broad range of BB and ABB genotypes, and a 0.35 kb band was limited to only one BB (K6) and one ABB genotype (K20). The Me6/Em8 primer produced a 0.25 kb band consistently found in all BB and ABB genotypes. It also amplified a 0.80 kb band unique to the cooking bananas, while a 0.30 kb band showed an unclear distribution. These genome-specific bands serve as useful molecular markers for distinguishing *Musa* genotypes and elucidating the genomic composition of ABB triploid hybrids in Thailand.

### 3.4 Genetic relationships among Thai banana genotypes

The genetic relationships among 28 Thai banana genotypes, including *M. acuminata* (AA), *M. balbisiana* (BB), and *Musa x paradisiaca* (ABB), were evaluated based on DNA fingerprint data generated from 13 RAPD and 28 SRAP markers. Using the Nei and Li similarity coefficient, genetic similarity values derived from RAPD data ranged from 0.337 to 1.000, with an average of 0.741 (Table 5). The lowest similarity (0.337) was observed between ‘Kluai Pa-Prae’ (AA; K1) and both ‘Kluai Tani Isan’ (BB; K5) and ‘Kluai Tani Dam’ (BB; K7). In contrast, the highest similarity (1.000) was detected between ‘Kluai Tani Isan’ (K5) and ‘Kluai Tani Dam’ (K7), as well as among ‘Kluai Namwa Dam’ (ABB; K8), ‘Kluai Namwa Pakchong 50’ (ABB; K12), and ‘Kluai Namwa Khom’ (ABB; K13), and between ‘Kluai Namwa Ubon’ (ABB; K9) and ‘Kluai Namwa Ngern’ (ABB; K10).

**Table 5:** Genetic similarity matrix of 28 *Musa* cultivars based on Nei and Li coefficients, with RAPD data shown in the upper triangular cells and SRAP data in the lower triangular cells.

	**K1**	**K2**	**K3**	**K4**	**K5**	**K6**	**K7**	**K8**	**K9**	**K10**	**K11**	**K12**	**K13**	**K14**	**K15**	**K16**	**K17**	**K18**	**K19**	**K20**	**K21**	**K22**	**K23**	**K24**	**K25**	**K26**	**K27**	**K28**
**K1**		0.565	0.584	0.571	0.337	0.352	0.337	0.387	0.391	0.391	0.404	0.387	0.387	0.400	0.404	0.392	0.408	0.404	0.433	0.431	0.409	0.400	0.387	0.358	0.371	0.424	0.424	0.424
**K2**	0.571		0.674	0.644	0.396	0.351	0.396	0.424	0.429	0.429	0.420	0.424	0.424	0.436	0.420	0.447	0.442	0.440	0.485	0.500	0.447	0.436	0.465	0.436	0.447	0.514	0.514	0.514
**K3**	0.611	0.789		0.943	0.388	0.383	0.388	0.458	0.463	0.463	0.454	0.458	0.458	0.490	0.495	0.480	0.475	0.454	0.520	0.514	0.418	0.449	0.458	0.429	0.440	0.529	0.529	0.529
**K4**	0.621	0.785	0.974		0.366	0.360	0.366	0.418	0.422	0.422	0.413	0.418	0.418	0.430	0.435	0.421	0.417	0.413	0.505	0.460	0.419	0.409	0.418	0.387	0.400	0.495	0.495	0.495
**K5**	0.306	0.299	0.266	0.262		0.900	1.000	0.863	0.851	0.851	0.835	0.863	0.863	0.846	0.854	0.868	0.841	0.835	0.736	0.775	0.804	0.846	0.843	0.865	0.887	0.741	0.722	0.722
**K6**	0.303	0.320	0.312	0.292	0.883		0.900	0.878	0.887	0.887	0.848	0.878	0.878	0.880	0.889	0.863	0.835	0.828	0.725	0.748	0.774	0.840	0.837	0.840	0.863	0.692	0.673	0.673
**K7**	0.302	0.304	0.270	0.258	0.988	0.897		0.863	0.851	0.851	0.835	0.863	0.863	0.846	0.854	0.868	0.841	0.835	0.736	0.775	0.804	0.846	0.843	0.865	0.887	0.741	0.722	0.722
**K8**	0.408	0.469	0.447	0.423	0.727	0.707	0.741		0.990	0.990	0.970	1.000	1.000	0.961	0.951	0.962	0.933	0.951	0.750	0.771	0.884	0.961	0.940	0.941	0.962	0.717	0.698	0.698
**K9**	0.408	0.469	0.447	0.423	0.727	0.707	0.741	1.000		1.000	0.960	0.990	0.990	0.970	0.960	0.951	0.923	0.940	0.757	0.759	0.872	0.951	0.929	0.931	0.951	0.724	0.705	0.705
**K10**	0.411	0.465	0.450	0.426	0.732	0.705	0.739	0.993	0.993		0.960	0.990	0.990	0.970	0.960	0.951	0.923	0.940	0.757	0.759	0.872	0.951	0.929	0.931	0.951	0.724	0.705	0.705
**K11**	0.417	0.464	0.449	0.425	0.737	0.710	0.743	0.990	0.990	0.997		0.970	0.970	0.932	0.922	0.933	0.962	0.980	0.743	0.745	0.896	0.971	0.951	0.932	0.952	0.692	0.673	0.692
**K12**	0.412	0.467	0.452	0.428	0.727	0.700	0.734	0.990	0.990	0.996	0.993		1.000	0.961	0.951	0.962	0.933	0.951	0.750	0.771	0.884	0.961	0.940	0.941	0.962	0.717	0.698	0.698
**K13**	0.411	0.465	0.450	0.426	0.732	0.705	0.739	0.993	0.993	1.000	0.997	0.996		0.961	0.951	0.962	0.933	0.951	0.750	0.771	0.884	0.961	0.940	0.941	0.962	0.717	0.698	0.698
**K14**	0.409	0.471	0.449	0.425	0.729	0.702	0.736	0.997	0.997	0.997	0.993	0.993	0.997		0.990	0.981	0.953	0.913	0.792	0.775	0.845	0.923	0.922	0.923	0.943	0.759	0.741	0.741
**K15**	0.409	0.471	0.449	0.425	0.722	0.702	0.736	0.997	0.997	0.990	0.986	0.993	0.990	0.993		0.971	0.943	0.902	0.800	0.782	0.833	0.913	0.911	0.913	0.933	0.766	0.748	0.748
**K16**	0.411	0.465	0.450	0.426	0.732	0.705	0.739	0.993	0.993	1.000	0.997	0.996	1.000	0.997	0.990		0.972	0.933	0.778	0.796	0.869	0.943	0.942	0.943	0.963	0.745	0.727	0.727
**K17**	0.411	0.465	0.450	0.426	0.732	0.705	0.739	0.993	0.993	1.000	0.997	0.996	1.000	0.997	0.990	1.000		0.962	0.771	0.772	0.880	0.953	0.952	0.935	0.954	0.721	0.703	0.721
**K18**	0.416	0.448	0.425	0.417	0.760	0.719	0.766	0.946	0.946	0.945	0.949	0.942	0.945	0.949	0.942	0.945	0.945		0.724	0.745	0.917	0.971	0.951	0.932	0.952	0.673	0.654	0.673
**K19**	0.438	0.418	0.416	0.390	0.751	0.763	0.758	0.783	0.783	0.781	0.785	0.777	0.781	0.778	0.778	0.781	0.781	0.771		0.743	0.747	0.736	0.750	0.736	0.741	0.927	0.909	0.927
**K20**	0.417	0.458	0.473	0.457	0.697	0.708	0.705	0.761	0.761	0.759	0.763	0.762	0.759	0.756	0.763	0.759	0.759	0.736	0.807		0.731	0.757	0.752	0.757	0.779	0.765	0.748	0.748
**K21**	0.405	0.453	0.437	0.420	0.727	0.708	0.742	0.969	0.969	0.968	0.972	0.965	0.968	0.965	0.965	0.968	0.968	0.928	0.777	0.747		0.887	0.884	0.845	0.869	0.693	0.673	0.693
**K22**	0.406	0.454	0.438	0.422	0.722	0.703	0.737	0.972	0.972	0.972	0.975	0.968	0.972	0.968	0.968	0.972	0.972	0.931	0.772	0.743	0.989		0.961	0.962	0.962	0.704	0.685	0.685
**K23**	0.422	0.454	0.424	0.408	0.765	0.716	0.771	0.949	0.949	0.949	0.952	0.945	0.949	0.952	0.946	0.949	0.949	0.990	0.769	0.734	0.925	0.928		0.941	0.942	0.698	0.679	0.698
**K24**	0.408	0.448	0.424	0.400	0.764	0.738	0.770	0.959	0.959	0.958	0.962	0.955	0.958	0.962	0.955	0.958	0.958	0.939	0.783	0.732	0.934	0.937	0.949		0.981	0.722	0.704	0.704
**K25**	0.409	0.471	0.449	0.425	0.729	0.702	0.736	0.997	0.997	0.997	0.993	0.993	0.997	1.000	0.993	0.997	0.997	0.949	0.778	0.756	0.965	0.968	0.952	0.962		0.727	0.709	0.709
**K26**	0.430	0.440	0.424	0.422	0.744	0.740	0.751	0.747	0.747	0.738	0.743	0.734	0.738	0.743	0.743	0.738	0.738	0.751	0.876	0.778	0.748	0.736	0.748	0.754	0.743		0.982	0.982
**K27**	0.430	0.440	0.424	0.422	0.744	0.740	0.751	0.747	0.747	0.738	0.743	0.734	0.738	0.743	0.743	0.738	0.738	0.751	0.876	0.778	0.748	0.736	0.748	0.754	0.743	1.000		0.982
**K28**	0.430	0.440	0.424	0.422	0.744	0.740	0.751	0.747	0.747	0.738	0.743	0.734	0.738	0.743	0.743	0.738	0.738	0.751	0.876	0.778	0.748	0.736	0.748	0.754	0.743	1.000	1.000	

For SRAP data, genetic similarity values ranged from 0.258 to 1.000, with an average of 0.738 (Table 5). The lowest similarity (0.258) occurred between ‘Kluai Khai Kamphaeng Phet’ (AA; K4) and ‘Kluai Tani Dam’ (BB; K7), whereas the highest similarity (1.000) was observed between several ABB genotypes, including ‘Kluai Namwa Dam’ (K8) and ‘Kluai Namwa Ubon’ (K9), among ‘Kluai Namwa Ngern’ (K10), ‘Kluai Namwa Khom’ (K13), ‘Kluai Namwa Nuan’ (K16), and ‘Kluai Namwa Sai Dam’ (K17), as well as between ‘Kluai Namwa Phrarachthan’ (K14) and ‘Kluai Namwa Tanao Sri’ (K25), and among the three ‘Kluai Hak Muk’ samples (K26–K28).

Based on the combined RAPD and SRAP data, genetic similarity coefficients ranged from 0.288 to 0.997, with an average of 0.739 (Supplementary Table S1). The lowest similarity (0.288) was observed between ‘Kluai Khai Kamphaeng Phet’ (K4) and ‘Kluai Tani Dam’ (K7), while the highest (0.997) was found between ‘Kluai Namwa Dam’ (K8) and ‘Kluai Namwa Ubon’ (K9).

### 3.5 Cluster analysis of Thai banana genotypes

**Fig. 2:**
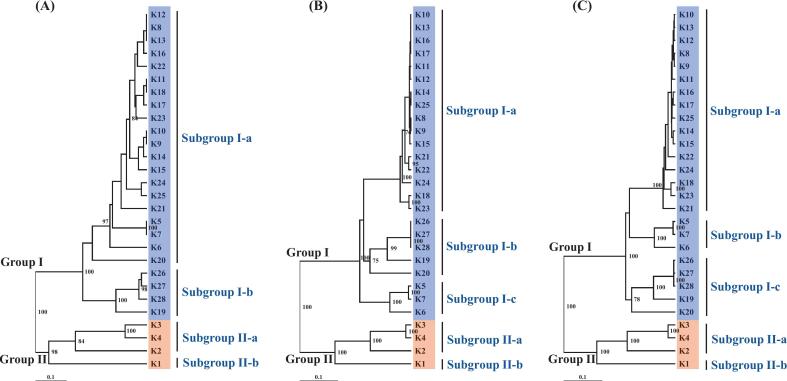
UPGMA dendrogram illustrating the genetic relationships among 28 *Musa* cultivars based on genetic similarity coefficients calculated from 13 RAPD primers (A), 28 SRAP primer combinations (B), and combined RAPD and SRAP data sets (C).

In this study, dendrograms were generated using the UPGMA clustering method based on RAPD, SRAP, and combined RAPD–SRAP data. Using RAPD data, the 28 Thai banana genotypes were grouped into two major clusters: Group I (comprising ABB and BB genome groups) and Group II (AA genome group). Each major group was further divided into two subgroups, designated as I-a, I-b and II-a, II-b, respectively (Fig. 2A).

Based on SRAP data, UPGMA clustering similarly divided the 28 Thai banana genotypes into two main groups (I and II) (Fig. 2B). Group I was further separated into three subgroups (I-a, I-b, and I-c), while Group II was divided into two subgroups (II-a and II-b).

The dendrogram constructed from the combined RAPD and SRAP data also grouped the 28 genotypes into two principal clusters (I and II) (Fig. 2C). Group I was subdivided into three subgroups: I-a, which included 16 ‘Kluai Namwa’ (ABB) genotypes; I-b, which comprised three ‘Kluai Tani’ (BB) genotypes; and I-c, consisting of five cooking banana (ABB) genotypes. Group II, corresponding to the AA genomic group, was divided into two subgroups (II-a and II-b). Bootstrap analysis supported the overall clustering pattern, with most nodes showing values above 70 %, although lower values in certain subgroups reflected internal genetic variability.

**Fig. 3:**
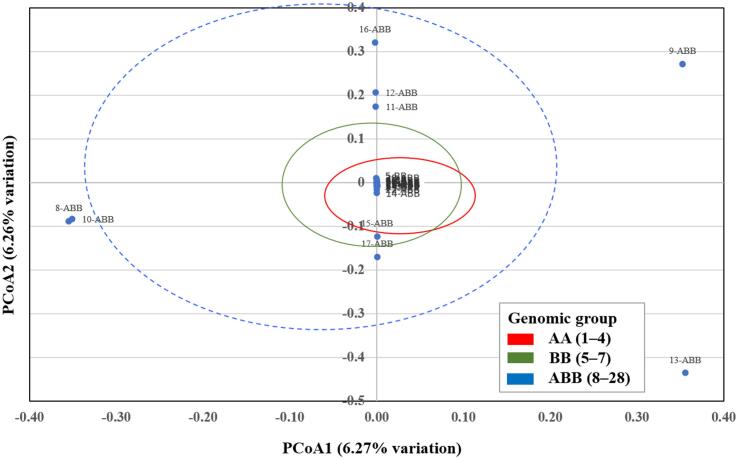
Principal Coordinates Analysis (PCoA) of *Musa* genotypes based on RAPD and SRAP markers. The analysis was based on a combined binary data matrix from 13 RAPD primers and 28 SRAP primer combinations. Samples are labeled by number (Table 1) and genomic group: AA (1–4, red), BB (5–7, green), and ABB (8–28, blue). Dashed ellipses enclose ABB genotypes, while circles highlight the smaller AA and BB groups. The clustering pattern is consistent with the UPGMA dendrogram (Fig. 2), confirming the genetic relationships and population structure.

To further validate the clustering patterns obtained by UPGMA, a Principal Coordinates Analysis (PCoA) was conducted using the combined RAPD and SRAP marker dataset. The first two coordinates explained 6.27 % (PCoA1) and 6.26 % (PCoA2) of the total genetic variation, respectively, accounting for a cumulative 12.53 % (Fig. 3). The PCoA plot revealed a grouping pattern consistent with the UPGMA dendrogram (Fig. 2). Genotypes belonging to the AA group (samples 1–4) and the BB group (samples 5–7) clustered separately and were enclosed by circles due to their small sample sizes, whereas the ABB group (samples 8–28) formed a broader cluster enclosed by a dashed ellipse. This independent ordination analysis confirms the genetic relationships and population structure revealed by the cluster analysis. In addition to the cluster-based analysis of individual genotypes, a genome-level comparison was performed to better understand the genetic differentiation within and between the major *Musa* genome groups (AA, BB, and ABB).

### 3.6 Comparative genetic similarity among Musa genomic groups

To further evaluate genome-level differentiation, average genetic similarity coefficients were calculated both within and between the AA, BB, and ABB genome groups (Table 6). The AA group exhibited moderate to high intra-group similarity, ranging from 0.570 to 0.966 (mean = 0.709), reflecting substantial genetic diversity among the diploid cultivars. In contrast, the BB group showed very high intra-group similarity (0.888–0.991; mean = 0.926), indicating a genetically uniform group with limited variation. The ABB triploid group displayed high overall similarity (0.722–0.997; mean = 0.878), although a certain degree of genetic variation was still observed among its cultivars.

**Table 6:** Comparative genetic similarity within and between *Musa* genome groups.

**Genome group compared**	**Range of genetic similarity (Coefficient)**	**Average**	**Interpretation**
Within AA	0.570–0.966	0.709	Moderate to high genetic similarity, indicating a diverse group with some closely related genotypes.
Within BB	0.888–0.991	0.926	Very high similarity, suggesting a genetically uniform group with minimal variation.
Within ABB	0.722–0.997	0.878	High similarity among triploid genotypes, with some variation observed among cultivars.
AA vs. BB	0.288–0.332	0.313	Very low similarity, reflecting the distinct genetic backgrounds of diploid AA and BB groups.
AA vs. ABB	0.394–0.485	0.436	Low to moderate similarity, as ABB triploids share genetic contributions from both AA and BB genomes but remain more distinct from AA.
BB vs. ABB	0.720–0.797	0.757	Moderate to high similarity, indicating a strong genetic contribution of the BB genome to ABB triploids.

Pairwise comparisons between genome groups revealed distinct patterns of relatedness. The lowest genetic similarity was observed between AA and BB genotypes (0.288–0.332; mean = 0.313), highlighting their divergent genomic origins. Comparisons between AA and ABB groups showed low to moderate similarity (0.394–0.485; mean = 0.436), consistent with the partial contribution of the AA genome to the ABB triploid lineage, alongside accumulated divergence. In contrast, the BB and ABB groups exhibited moderate to high similarity (0.720–0.797; mean = 0.757), underscoring the stronger genetic influence of the BB genome in the ABB group. These findings support the hypothesis that ABB triploid bananas share a closer genomic affinity with BB genotypes than with AA, aligning with their known hybrid origin.

### 3.7 Genetic stability assessment of ‘Kluai Namwa Mali-Ong’ plantlets

**Fig. 4:**
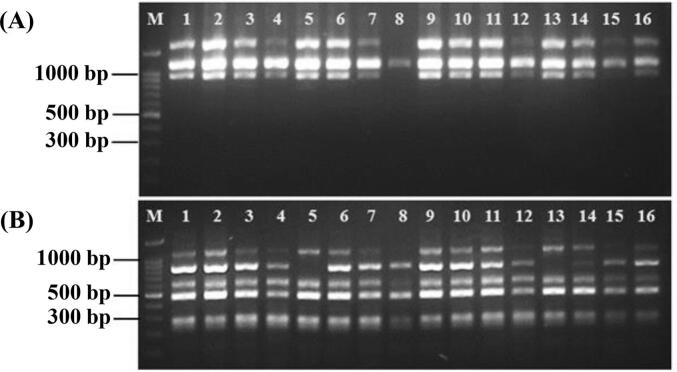
DNA amplification profiles of 16 *Musa* ‘Kluai Namwa Mali-Ong’ samples generated with RAPD primer S33 (A) and SRAP primer combination Me1/Em6 (B). Lane M indicates the 100 bp Plus DNA ladder (OneMARK, BIO-HELIX, Taiwan). Cultivar numbers correspond to those listed in Table 1 (nos. 11 and 29–43).

Genetic stability of 16 *Musa* ‘Kluai Namwa Mali-Ong’ banana plantlets was assessed using 14 markers, comprising seven RAPD and seven SRAP primer pairs. A total of 71 DNA bands were amplified, with each marker generating between three (S25, S33) and seven bands (S11, Me6/Em7), averaging 5.07 bands per marker (Table 7). Of these, 49 bands were polymorphic and 22 monomorphic, resulting in an average of 3.50 polymorphic bands per primer. Representative RAPD (S33) and SRAP (Me1/Em6) profiles of 16 ‘Kluai Namwa Mali-Ong’ cultivars are shown in Fig. 4.

**Table 7:** Characteristics of 14 primers used for DNA amplification in 16 *Musa* ‘Kluai Namwa Mali-Ong’ cultivars, including allele size range, percentage of polymorphism, PIC values, effective multiplex ratio (EMR), and marker index (MI).

**No.**	**Primer**	**Allele size range (kb)**	**NA**	**NP**	**PPB (%)**	**PIC**	**EMR**	**MI**
1	S11	0.35–1.70	7	4	57.14	0.17	4	0.68
2	S12	0.55–1.20	5	5	100.0	0.12	5	0.60
3	S18	0.45–1.50	4	3	75.00	0.18	3	0.54
4	S25	0.90–1.20	3	3	100.0	0.40	3	1.20
5	S28	0.35–1.50	5	4	80.00	0.29	4	1.16
6	S33	1.00–1.70	3	2	66.67	0.15	2	0.30
7	S38	0.30–2.00	5	4	80.00	0.26	4	1.04
8	Me1/Em6	0.20–1.00	5	2	40.00	0.11	2	0.22
9	Me4/Em8	0.15–0.70	5	4	80.00	0.16	4	0.64
10	Me5/Em1	0.20–1.20	6	3	50.00	0.12	3	0.36
11	Me6/Em2	0.20–1.00	5	3	60.00	0.13	3	0.39
12	Me6/Em3	0.35–1.40	5	3	60.00	0.17	3	0.51
13	Me6/Em4	0.10–1.20	6	3	50.00	0.17	3	0.51
14	Me6/Em7	0.18–1.20	7	6	85.71	0.32	6	1.92
	**Total**	0.10–2.00	**71**	**49**	−	−	**49**	**10.07**
	**Average**		**5.07**	**3.50**	**69.01**	**0.20**	**3.50**	**0.72**

**Note:** NA, total number of amplified fragments; NP, total number of polymorphic fragments; PPB, Percentage of polymorphic fragments (%); PIC, Polymorphism information content; EMR, Effective multiplex ratio; MI, Marker index.

**Fig. 5:**
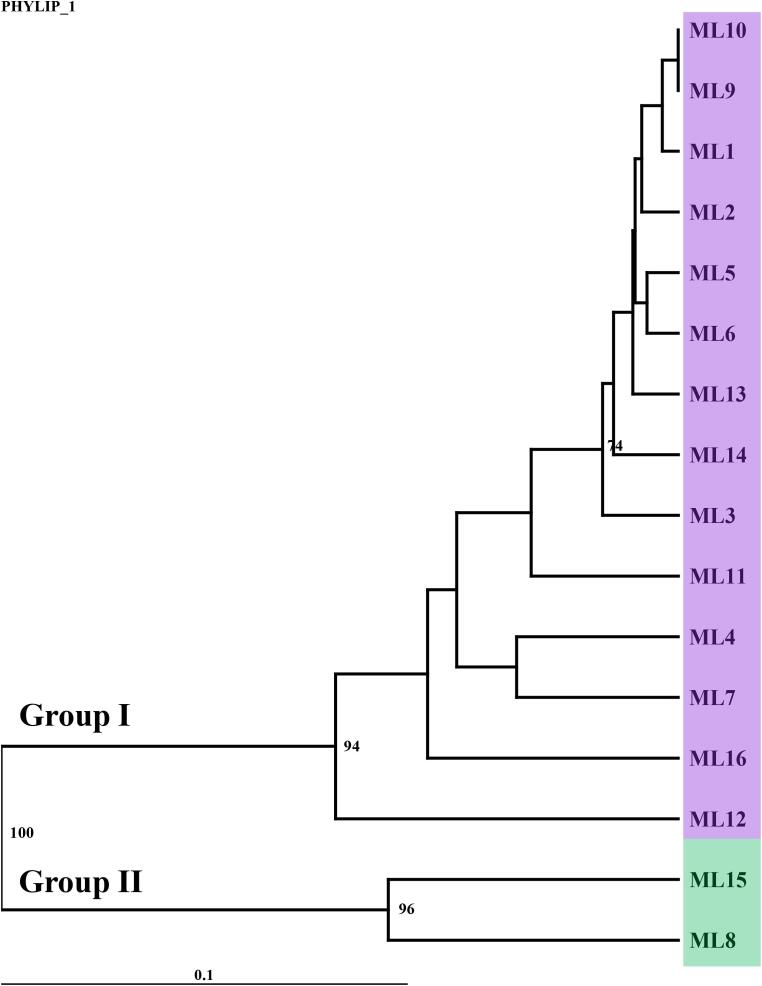
UPGMA dendrogram illustrating genetic relationships among 16 populations of *Musa × paradisiaca* ‘Kluai Namwa Mali-Ong’ (ABB group), based on genetic similarity coefficients derived from 7 RAPD primers and 7 SRAP primer combinations.

Genetic similarity coefficients among the ‘Mali-Ong’ samples ranged from 0.571 to 1.000, with a mean value of 0.858 (Table 8), indicating moderate to high genetic similarity. UPGMA cluster analysis grouped the samples into two major clusters (Fig. 5). Group I consisted primarily of samples from farmer cultivation areas and tissue-culture plantlets obtained from nurseries in Phitsanulok Province and other regions of Thailand, while Group II included samples from Samut Songkhram Province (ML8) and Pathum Thani Province (ML15), indicating a possible clonal variation within ‘Mali-Ong’ subvarieties.

**Table 8:** Genetic similarity matrix for 16 populations of *Musa × paradisiaca* ‘Kluai Namwa Mali-Ong’ (ABB Group), based on Nei and Li coefficients derived from RAPD and SRAP marker data.

	**ML1**	**ML2**	**ML3**	**ML4**	**ML5**	**ML6**	**ML7**	**ML8**	**ML9**	**ML10**	**ML11**	**ML12**	**ML13**	**ML14**	**ML15**	**ML16**
**ML1**																
**ML2**	0.977															
**ML3**	0.961	0.969														
**ML4**	0.883	0.878	0.909													
**ML5**	0.976	0.969	0.969	0.876												
**ML6**	0.977	0.970	0.969	0.894	0.985											
**ML7**	0.924	0.902	0.933	0.920	0.917	0.918										
**ML8**	0.680	0.680	0.694	0.703	0.673	0.680	0.733									
**ML9**	0.992	0.985	0.969	0.876	0.984	0.985	0.917	0.694								
**ML10**	0.992	0.985	0.969	0.876	0.984	0.985	0.917	0.694	1.000							
**ML11**	0.928	0.938	0.921	0.840	0.921	0.922	0.847	0.646	0.937	0.937						
**ML12**	0.814	0.828	0.860	0.916	0.825	0.828	0.849	0.786	0.825	0.825	0.804					
**ML13**	0.976	0.969	0.952	0.857	0.984	0.969	0.898	0.688	0.984	0.984	0.919	0.821				
**ML14**	0.968	0.961	0.944	0.864	0.960	0.961	0.889	0.695	0.976	0.976	0.927	0.829	0.976			
**ML15**	0.630	0.611	0.624	0.674	0.602	0.611	0.682	0.857	0.624	0.624	0.571	0.709	0.615	0.622		
**ML16**	0.904	0.881	0.862	0.826	0.879	0.881	0.833	0.721	0.897	0.897	0.842	0.784	0.895	0.920	0.691

## 4 Discussion

### 4.1 Genetic diversity of Thai banana genotypes revealed by RAPD and SRAP markers

In this study, both RAPD and SRAP markers proved effective in revealing the genetic distinctiveness of Thai banana genotypes, with high polymorphism levels of 93.58 % and 92.45 %, respectively. These results reflect substantial genetic variability among the 28 *Musa* genotypes analyzed, encompassing representatives of the AA, BB, and ABB genomic groups. Notably, despite the often similar and overlapping morphological features observed among ABB genotypes, the molecular data clearly demonstrate extensive genotypic diversity within this group. The high marker index (MI) and effective multiplex ratio (EMR) values further validate the efficiency of the selected primers in detecting polymorphisms, underscoring their value for genotype discrimination and germplasm characterization.

Among the two marker types, SRAP markers were particularly informative due to their ability to target open reading frames (ORFs), enabling the detection of functionally relevant genetic variation. These findings are consistent with those of Cruz-Cárdenas et al.^30^ who reported the usefulness of SRAP markers in *Musa* genotyping. Similarly, other studies employing diverse molecular systems, including RAPD, AFLP, SSR, ISSR, SRAP, and SCoT, have reported considerable genetic diversity in triploid *Musa* accessions,^13^, ^16^, ^20^, ^31^, ^32^ supporting the high polymorphism observed in our analysis.

Although significant genetic variation was detected at the molecular level, these differences did not consistently align with morphological traits. Subvarieties such as ‘Mali-Ong’, ‘Pakchong 50’, ‘Kab Khao’, and ‘Suan’ share similar general morphological characteristics, including bunch architecture, fruit shape, and peel coloration (Supplementary Table S2), making field identification challenging. Some accessions exhibit distinguishing features, such as the reddish pseudostem base in ‘Kluai Namwa Suan’, bluish peel in ‘Kluai Namwa Khieo’, or orange flesh in ‘Kluai Hak Muk’, but these traits are often subtle and influenced by environmental conditions like soil type, elevation, and cultivation practices. This weak genotype–phenotype correlation underscores the limitations of relying solely on morphological descriptors and highlights the importance of molecular markers for accurate classification within the ABB group.

In this context, the integration of molecular markers, particularly RAPD and SRAP, offers a powerful complementary approach for discriminating closely related genotypes. These tools enhance genetic resolution, reveal cryptic diversity, and provide a more objective basis for germplasm conservation, clonal identification, and cultivar improvement. The molecular insights gained from this study reaffirm the value of these markers and lay a strong foundation for future breeding programs targeting trait enhancement in Thai triploid bananas.

### 4.2 Genome-specific markers and their utility in banana genotyping

The identification of genome-specific DNA bands among Thai banana genotypes highlights the genetic complexity of ABB triploid bananas and their diploid progenitors. Both RAPD and SRAP markers proved effective in distinguishing the A and B genome contributions, corroborating earlier studies.^16^, ^33^ For instance, B genome-specific bands such as RAPD-S1 (0.50 kb) and SRAP-Me1/Em4 (0.80 kb) were consistently detected in BB diploid accessions and ‘Bluggoe’ cooking bananas, confirming the strong presence of the B genome in these genotypes. This observation aligns with genomic studies linking specific DNA sequences and structural elements to *M. balbisiana*.^34^ In contrast, A genome-specific bands, including RAPD-S6 (1.80 kb) and SRAP-Me2/Em5 (0.40–0.60 kb), were predominantly found in AA diploids and Pisang Awak-type ABB genotypes. This supports their derivation from *M. acuminata*, consistent with prior reports on the A genome origin of triploid bananas.^35^ Additionally, polymorphic bands produced by SRAP-Me1/Em6 in both BB and ABB groups further underscore the contribution of the B genome to ABB genotypes.

Interestingly, the SRAP-Me6/Em8 primer combination amplified a shared band in both AA and BB genotypes, suggesting the presence of conserved or homologous loci across the A and B genomes. This may reflect ancestral sequences retained from a common progenitor or indicate convergent evolution of certain genomic regions. Unique bands observed exclusively in the ‘Bluggoe’ cooking bananas (K19, K20, K26–K28), such as RAPD-S7 (2.50 kb) and SRAP-Me6/Em8 (0.80 kb), may point to lineage-specific selection or introgression events, indicating distinct genetic divergence within this subgroup. In contrast, Pisang Awak accessions (K8–K18, K21–K25) displayed A genome-associated bands characteristic of dessert-type bananas, reinforcing their close genetic relationship to *M. acuminata* and supporting previous findings on genome composition in triploid bananas.^36^, ^37^

Overall, the genome-specific markers used in this study proved to be reliable tools for determining genome composition and offered valuable insights into inheritance patterns in triploid hybrids. The consistent detection of both A and B genome-specific bands in ABB genotypes such as ‘Kluai Namwa’ confirms their hybrid origin and supports genome-based classification, which is an essential component in banana breeding and conservation efforts.^20^, ^38^ Unlike morphological traits, which are often influenced by environmental factors, molecular markers provide stable and reproducible approaches for genome identification, varietal authentication, and genebank management. However, the study faced limitations in differentiating closely related ‘Kluai Namwa’ subvarieties. The limited resolution and dominant nature of RAPD and SRAP markers, which may not detect subtle genetic differences, are more likely to explain this difficulty than low genetic variability. Despite the high morphological diversity observed among ‘Kluai Namwa’ subvarieties, including differences in fruit shape, size, and color, these traits may result from minor genetic changes in regulatory regions, differential gene expression, or epigenetic modifications.^39^ Such variations are often undetectable with dominant markers that randomly sample non-coding or non-functional regions of the genome.^19^ Therefore, higher-resolution and co-dominant marker systems such as SSRs, ISSRs, or SNPs are recommended for future studies to enable finer-scale genotyping and to better capture the genetic basis of morphological variation.^5^, ^40^

### 4.3 Insights into genetic similarity and differentiation among Thai banana genotypes

The genetic similarity patterns among the 28 Thai banana genotypes reveal significant genome-level structure, offering insights into their evolutionary origins, domestication history, and potential applications in genetic improvement. Intragroup similarity within the AA, BB, and ABB groups varied notably, reflecting the influence of different domestication pathways, parental genome contributions, and propagation practices. Among AA diploids, a moderate mean similarity coefficient of 0.709 indicates considerable genetic diversity. This variation likely reflects historical selection under diverse agroecological conditions and the accumulation of somatic mutations through long-term vegetative propagation. For instance, ‘Kluai Pa-Prae’ (K1) was genetically distinct from other accessions, including both AA and BB types, suggesting a unique genetic background. Such diversity among diploids is a valuable resource for breeding programs aiming to enhance traits like fruit quality, disease resistance, and abiotic stress tolerance.^38^ In contrast, BB diploid genotypes displayed high genetic uniformity (mean = 0.926), with nearly identical profiles observed between ‘Kluai Tani Isan’ (K5) and ‘Kluai Tani Dam’ (K7). This high similarity may reflect the narrow genetic base of cultivated *M.balbisiana* in Thailand, likely due to limited incorporation of wild accessions.^41^ Despite this uniformity, BB types remain crucial in breeding programs for their known contributions to vigor, drought tolerance, and disease resistance.^42^ However, the limited number of AA and BB samples in this study may not accurately reflect the greater diversity within these groups. Broader sampling of both cultivated and wild accessions is necessary to obtain a more comprehensive understanding of the genetic landscape of diploid bananas in Thailand.

ABB triploid genotypes, including the widely cultivated ‘Kluai Namwa’ types, also exhibited high intragroup similarity (mean = 0.878). Notably, several cultivars such as ‘Kluai Namwa Dam’ (K8), ‘Kluai Namwa Ubon’ (K9), and ‘Kluai Namwa Ngoen’ (K10) shared a similarity coefficient of 1.000, indicating extremely low genetic differentiation among them. This high uniformity may not solely reflect clonal propagation but could also result from a narrow genetic base at the time of domestication and limited incorporation of genetically diverse parental lines in breeding or selection processes.^41^, ^43^ In triploid bananas, where sexual reproduction is rare or absent, the initial genetic composition established during early hybridization events can remain largely unchanged, particularly when derived from a small number of founding clones.^8^ Additionally, the dominant molecular markers employed (RAPD and SRAP) may not detect minor allelic variations or structural differences, potentially underestimating the true extent of genetic diversity.^1^, ^16^ This underscores the importance of employing higher-resolution and co-dominant marker systems such as SSRs or SNP arrays to uncover finer-scale variation that could be linked to phenotypic traits, regional adaptation, or somatic mutations in clonally propagated crops.^8^, ^39^ Similarly, the tight clustering of the three ‘Kluai Hak Muk’ accessions (K26–K28) suggests a shared clonal origin, with subsequent selection likely driven by localized preferences for fruit characteristics or agronomic performance. Intergroup comparisons further clarified the hybrid nature of ABB genotypes. The lowest similarity was observed between AA and BB groups (mean = 0.313), consistent with their divergent species origins (*M. acuminata* vs. *M. balbisiana*). Comparisons between ABB and AA (mean = 0.436) were lower than those between ABB and BB (mean = 0.757), suggesting a stronger retention of *M. balbisiana* alleles in ABB triploids. This pattern supports the concept of B genome dominance in interspecific hybrids, as indicated in cytogenetic and molecular studies.^1^, ^44^

These findings have practical implications for both banana breeding and conservation strategies. The high similarity among Thai ABB genotypes highlights the need to expand the genetic base of triploids through the introduction of diverse ABB or AAB/AB hybrids. Meanwhile, genetically distinct AA cultivars like ‘Kluai Pa-Prae’ (K1) and ‘Kluai Khai Kamphaeng Phet’ (K4) may serve as valuable donors of novel alleles for trait improvement. Overall, the results reinforce the importance of genome-based classification frameworks and confirm the utility of molecular markers for genotypic verification, varietal authentication, and efficient germplasm management.

### 4.4 Cluster patterns and genomic relationships among Musa genomic groups

Cluster analyses based on RAPD, SRAP, and combined marker datasets consistently grouped the 28 Thai banana genotypes into two major clusters, reflecting their underlying genomic constitutions (AA versus ABB/BB). This outcome affirms the utility of marker-based classification in *Musa* genetic studies.^13^, ^45^. Group II consisted exclusively of AA diploid genotypes, clearly separated from Group I, which included both ABB triploid and BB diploid genotypes. This separation mirrors the pronounced genomic divergence between *M. acuminata* (A genome) and *M. balbisiana* (B genome) lineages.^42^

The combined RAPD–SRAP dendrogram provided enhanced resolution, allowing further subdivision within Group I into three biologically meaningful subgroups: Subgroup I-a comprised 16 ‘Kluai Namwa’ genotypes (ABB), reflecting high genetic similarity and clonal relatedness. The tight clustering of accessions such as ‘Kluai Namwa Dam’, ‘Kluai Namwa Ubon’, and ‘Kluai Namwa Khom’ supports their likely derivation from a common ancestral hybrid lineage, potentially differentiated through somatic mutation or localized selection.^46^, ^47^ Subgroup I-b included the three BB genotypes (‘Kluai Tani’ types), highlighting the genetic distinctness of *M. balbisiana*-derived accessions and their role as ancestral donors in triploid hybrids.^38^, ^41^ Subgroup I-c contained five other ABB cooking-type bananas, showing intermediate divergence within the triploid group and suggesting possible independent hybrid origins or distinct domestication pathways. Group II (AA) was further separated into subgroups II-a and II-b, reflecting subtle genetic differences among diploid genotypes such as ‘Kluai Hom Champa’ and ‘Kluai Pa-Prae’. These patterns may reflect regional diversification, traditional propagation practices, or introductions from genetically distinct populations, as reported in previous studies of Thai banana germplasm. Notably, the consistent clustering of ABB triploids closer to BB diploids than to AA types reinforces the hybrid origin of ABB bananas. This pattern reflects the asymmetric genomic contribution in ABB genotypes, with two B genome sets and one A genome set, resulting in greater genetic affinity to *M. balbisiana*.^38^, ^42^

Overall, the congruence across RAPD, SRAP, and combined analyses, supported by strong bootstrap values, demonstrates the reliability of dominant molecular markers in elucidating genomic relationships among banana genome groups. These findings reinforce the use of DNA marker systems for genome-based classification and offer valuable guidance for breeding, germplasm conservation, and varietal authentication within Thai *Musa* collections.

### 4.5 Assessment of clonal variation and genetic stability in ‘Kluai Namwa Mali-Ong’ plantlets

The genetic integrity of ‘Namwa Mali-Ong’, a widely cultivated ABB banana cultivar in Thailand, was assessed using RAPD and SRAP markers. Results revealed moderate polymorphism among the 16 clonal accessions, with genetic similarity coefficients ranging from 0.571 to 1.000. Most samples exhibited high similarity, indicating a largely conserved genetic background despite their different geographic origins. Cluster analysis confirmed this pattern, placing the majority of accessions within a single dominant group. However, two plantlets, ML8 from Samut Songkhram and ML15 from Pathum Thani, formed a separate cluster, suggesting subtle genetic variation that may be attributed to somatic mutations, epigenetic changes, or micropropagation-induced variability. These findings align with previous research. Premjet et al.^13^ reported high molecular uniformity among ‘Namwa Mali-Ong’ clones, despite minor phenotypic differences in plant architecture. Similarly, Suvittawat et al.^48^ observed notable variability in vegetative traits among ‘Namwa’ accessions, while maintaining stable fruit characteristics. This suggests that while phenotypic plasticity may arise under different agroecological conditions or propagation methods, the core genome of ‘Namwa Mali-Ong’ remains relatively stable. The observed genetic stability, coupled with adaptability and desirable agronomic traits, underscores the potential of ‘Namwa Mali-Ong’ as a reliable cultivar for mass propagation, germplasm conservation, and future breeding programs. However, the detection of minor clonal divergence also highlights the need for regular genotypic monitoring in micropropagation systems to maintain cultivar integrity.

### 4.6 Implications for breeding and germplasm management

The molecular differentiation observed among the 28 Thai banana genotypes, particularly across genome groups AA, BB, and ABB, highlights the importance of genomic characterization in guiding targeted breeding efforts. The consistent clustering of ‘Kluai Namwa’ (ABB) genotypes into a well-defined subgroup reflects a genetically stable lineage that can be utilized for traits such as yield stability, adaptability, and disease resistance. In contrast, the genetic distinctness of ‘Kluai Tani’ (BB) and AA diploid genotypes presents valuable opportunities for broadening the genetic base through inter-genomic hybridization, especially for enhancing stress tolerance and resistance to pests and diseases.

The moderate polymorphism detected among ‘Kluai Namwa Mali-Ong’ clones points to low but detectable clonal variation, likely arising from somatic mutations, epigenetic modifications, or propagation practices. While this variation is limited, it may provide a useful basis for selecting locally adapted lines. At the same time, it underscores the need for genetic monitoring to prevent the accumulation of undesirable changes in clonally propagated material. Marker-assisted selection and routine genetic fingerprinting are therefore essential for ensuring cultivar fidelity in commercial propagation systems. With the narrow genetic base observed in ABB cultivars like ‘Kluai Namwa’, innovative approaches such as genome editing could complement traditional breeding by introducing targeted genetic variation without altering desirable traits.^49^, ^50^ This strategy holds potential for enhancing stress tolerance, disease resistance, and other agronomic traits while preserving clonal identity.

In terms of germplasm management, the clustering patterns that reflect geographic origin suggest that local propagation and selection practices shape the current genetic structure of banana cultivars. This reinforces the importance of documenting provenance and developing core collections that capture both genetic and regional diversity. For breeding programs, integrating molecular marker data with phenotypic traits will improve the identification of elite parental lines, enhance hybrid performance prediction, and support long-term conservation and utilization of *Musa* genetic resources in Thailand and the wider Southeast Asian region.

## 5 Conclusion

In summary, this study provides the first systematic molecular characterization of 43 Thai banana genotypes, including 28 diverse accessions and 16 ‘Kluai Namwa Mali-Ong’ plantlets, using RAPD and SRAP markers. Clear genetic differentiation was observed among AA, BB, and ABB groups, with the ABB ‘Kluai Namwa’ subvarieties forming a cohesive cluster and ‘Kluai Hak Muk’ distinguished by unique bands. The Mali-Ong plantlets showed high clonal uniformity (mean similarity = 0.858), confirming their reliability in propagation. However, the use of RAPD and SRAP as dominant markers limited the resolution of allelic diversity and heterozygosity. Future research incorporating co-dominant or high-resolution markers such as SSRs, SNPs, or genotyping-by-sequencing, combined with phenotypic and ecological data, will enable finer-scale assessments of genetic diversity and adaptation. Importantly, this study provides the first comprehensive molecular evidence of genetic differentiation and clonal stability in Thai ABB bananas, with particular emphasis on the commercially important ‘Kluai Namwa’ group. These findings offer essential insights to support germplasm conservation, cultivar authentication, and targeted breeding strategies.

## Declaration of Generative AI and AI-assisted technologies in the writing process

During the preparation of this work, the authors used ChatGPT to assist in drafting, revising, and polishing sections of the manuscript. After using this tool, the authors carefully reviewed and edited the content to ensure accuracy, clarity, and alignment with the study’s findings, and take full responsibility for the final content of the publication.

## CRediT authorship contribution statement

**Thanita Boonsrangsom:** Writing – review & editing, Writing – original draft, Visualization, Validation, Software, Resources, Project administration, Methodology, Investigation, Formal analysis, Data curation, Conceptualization. **Kawee Sujipuli:** Validation, Resources. **Duangporn Premjet:** Resources, Funding acquisition.

## Funding

This work was financially supported by the National Science, Research and Innovation Fund (NSRF) (Grant No. FRB660001/0179) and Naresuan University (Grant No. R2566B070).

## Declaration of competing interest

The authors declare that they have no known competing financial interests or personal relationships that could have appeared to influence the work reported in this paper.
