# Supplementary material for: Genetic differentiation of Pisang Awak subvarieties and genetic variation among ‘Mali-Ong’ plantlets in Thailand using RAPD and SRAP markers
Source: J Genet Eng Biotechnol. 2025 Oct 3;23(4):100577. doi: 10.1016/j.jgeb.2025.100577 (PMC12516043; doi:10.1016/j.jgeb.2025.100577)
Supplement: Supplementary Data 1 [file mmc1.pdf]

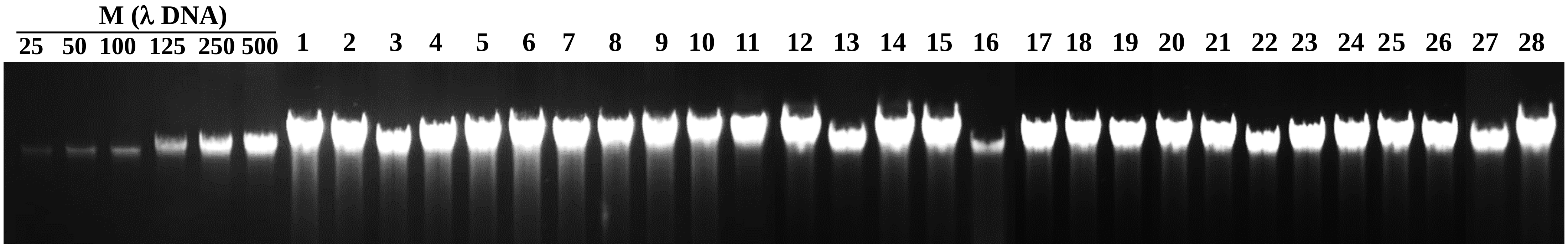

Supplementary Figure S1. Representative gel image showing the integrity of genomic DNA extracted from banana leaf samples. Lanes 1–28 contain 5 µL of genomic DNA isolated from *Musa* genotypes listed in Table 1. Lane M represents the λ DNA, with bands corresponding to 25, 50, 100, 125, 250, and 500 ng/µL. All samples exhibited intact, high-molecular-weight DNA with minimal degradation, confirming their suitability for subsequent RAPD and SRAP analyses.

Supplementary Table S1. Genetic similarity matrix of 28 *Musa* cultivars based on Nei and Li coefficients using combined RAPD and SRAP marker data

	**K1**	**K2**	**K3**	**K4**	**K5**	**K6**	**K7**	**K8**	**K9**	**K10**	**K11**	**K12**	**K13**	**K14**	**K15**	**K16**	**K17**	**K18**	**K19**	**K20**	**K21**	**K22**	**K23**	**K24**	**K25**	**K26**	**K27**	**K28**
**K1**																												
**K2**	0.570																											
**K3**	0.604	0.757																										
**K4**	0.608	0.748	0.966																									
**K5**	0.315	0.327	0.301	0.291																								
**K6**	0.316	0.329	0.332	0.311	0.888																							
**K7**	0.312	0.330	0.304	0.288	0.991	0.898																						
**K8**	0.402	0.457	0.450	0.422	0.764	0.753	0.774																					
**K9**	0.403	0.459	0.451	0.423	0.761	0.756	0.771	0.997																				
**K10**	0.406	0.456	0.454	0.425	0.765	0.754	0.770	0.992	0.995																			
**K11**	0.413	0.452	0.450	0.422	0.764	0.748	0.769	0.985	0.982	0.987																		
**K12**	0.406	0.456	0.454	0.425	0.765	0.749	0.770	0.992	0.990	0.995	0.987																	
**K13**	0.404	0.455	0.453	0.424	0.768	0.752	0.773	0.995	0.992	0.997	0.990	0.997																
**K14**	0.407	0.462	0.460	0.426	0.762	0.751	0.767	0.987	0.990	0.990	0.977	0.985	0.987															
**K15**	0.408	0.457	0.461	0.427	0.759	0.753	0.769	0.985	0.987	0.982	0.969	0.982	0.979	0.992														
**K16**	0.406	0.460	0.459	0.425	0.771	0.749	0.775	0.985	0.982	0.987	0.980	0.987	0.990	0.992	0.985													
**K17**	0.410	0.459	0.457	0.424	0.763	0.742	0.768	0.977	0.974	0.979	0.987	0.979	0.982	0.985	0.977	0.992												
**K18**	0.413	0.446	0.433	0.416	0.781	0.749	0.785	0.947	0.944	0.944	0.957	0.944	0.947	0.939	0.932	0.942	0.950											
**K19**	0.437	0.437	0.446	0.422	0.747	0.752	0.751	0.774	0.776	0.775	0.774	0.769	0.772	0.782	0.784	0.780	0.778	0.758										
**K20**	0.421	0.470	0.485	0.458	0.720	0.720	0.725	0.763	0.760	0.759	0.758	0.764	0.762	0.761	0.768	0.770	0.763	0.739	0.789									
**K21**	0.406	0.451	0.432	0.420	0.748	0.725	0.758	0.948	0.945	0.945	0.953	0.945	0.947	0.935	0.932	0.943	0.945	0.925	0.769	0.743								
**K22**	0.404	0.449	0.441	0.418	0.757	0.741	0.768	0.969	0.966	0.966	0.974	0.966	0.969	0.956	0.954	0.964	0.967	0.941	0.762	0.747	0.963							
**K23**	0.413	0.457	0.433	0.410	0.786	0.749	0.790	0.947	0.944	0.944	0.952	0.944	0.947	0.944	0.937	0.947	0.950	0.980	0.764	0.739	0.915	0.936						
**K24**	0.394	0.444	0.425	0.397	0.792	0.766	0.797	0.954	0.951	0.951	0.954	0.951	0.954	0.952	0.944	0.954	0.952	0.937	0.770	0.739	0.911	0.944	0.947					
**K25**	0.399	0.464	0.446	0.418	0.774	0.747	0.779	0.987	0.985	0.985	0.982	0.985	0.987	0.985	0.977	0.987	0.985	0.950	0.768	0.763	0.940	0.967	0.950	0.967				
**K26**	0.428	0.461	0.454	0.443	0.743	0.726	0.748	0.739	0.741	0.734	0.729	0.729	0.732	0.747	0.749	0.740	0.733	0.730	0.891	0.774	0.734	0.727	0.735	0.746	0.738			
**K27**	0.428	0.461	0.454	0.443	0.738	0.721	0.743	0.734	0.736	0.729	0.724	0.724	0.727	0.742	0.744	0.735	0.728	0.724	0.886	0.769	0.728	0.722	0.730	0.740	0.733	0.995		
**K28**	0.428	0.461	0.454	0.443	0.738	0.721	0.743	0.734	0.736	0.729	0.729	0.724	0.727	0.742	0.744	0.735	0.733	0.730	0.891	0.769	0.734	0.722	0.735	0.740	0.733	0.995	0.995	

Supplementary Table S2. Key morphological characteristics of *Musa × paradisiaca g*ermplasms (ABB Group) collected in Thailand

Subvariety Name	Pseudostem Color	Height (cm)	Bunch/Hand Count	Fruit Shape/Size	Fruit Skin (Raw/Ripe)	Flesh Color/Taste	Notable Features
Kluai Namwa Mali-Ong	Light green; varies between sub-strains	Medium-tall (350–450)	9–16 hands per bunch	Short, plump, tightly packed; irregular arrangement	Raw: Light/pale green<br>Ripe: Thin-skinned, deep yellow to creamy yellow	Creamy flesh, yellow core; sweet with slight tang	Two sub-strains: 'Kiew' (greener raw peel) and 'Khao' (creamier ripe peel); known distinct flavor
Kluai Namwa Pakchong 50	Pinkish green base with red blotches on inner sheath	Tall (>300)	Avg. 11.6 hands, 18.5 fruits/hand	Large, uniform; thick peel	Raw: Green<br>Ripe: Yellow	Firm yellow core; high sweetness (26.9% Brix)	High-yield hybrid; max yield 30.6 kg/plant; commercial variety
Kluai Namwa Nuan / Ngern	Light green with pink tinge	Medium (320–350)	7–9 hands per bunch; 12–14 fruits/hand	Angular unripe; rounder when ripe	Raw: Pale green-blue<br>Ripe: Pale yellow, thick peel	White flesh, yellow core; firm and mildly sweet	Pale fruit color; firm texture; larger bunches than typical Namwa
Kluai Namwa Dam	Medium green	Medium (280–300)	5–9 hands per bunch; 12–14 fruits/hand	Variable size; may contain seeds	Raw: Dull green<br>Ripe: Rust-speckled, turns brown/black	Yellowish-white; sticky, very sweet	Long shelf life (>2 weeks); peel color darkens with ripeness; high potassium
Kluai Namwa Khom	Light green	Short (150–180)	5–7 hands per bunch; 10–12 fruits/hand	Small; some thin/underdeveloped	Raw: Light green<br>Ripe: Pale yellow, thin peel	White to creamy flesh, yellow core; mild sweetness	Dwarf type; suitable for compact cultivation
Kluai Namwa Phrarachthan / Yak	-	-	Up to 7–8 kg per hand; ~100 kg per bunch	Very large fruits	Raw: Green<br>Ripe: Yellow	Firm and sweet, even when unripe	Extra-large size; firm, non-fibrous core
Kluai Namwa Sai Lueang	Yellowish green	Medium (<380)	7–9 hands per bunch	Plump when mature	Raw: Pale green<br>Ripe: Pale yellow, thick peel	Cream flesh, yellow core; soft, mildly sweet with slight sourness	High pulp color diversity (white/yellow/red); seedless
Kluai Namwa Khieo (Green Namwa)	Medium green	Medium (320–350)	9–11 hands per bunch; 12–16 fruits/hand	Angular when unripe, medium size	Raw: Dull green<br>Ripe: Yellow with green ridges	Yellow flesh, yellow/reddish core; sweet	May contain 2–3 flat seeds; rust-like speckles when ripe
Kluai Namwa Khao (White Namwa)	Light green	Medium-tall (400–450)	5–10 hands per bunch	Medium-sized	Raw: Green<br>Ripe: Yellow	Fluffy white flesh; whiter core; sweet	Tall plant with very light-colored flesh
Kluai Namwa Kab Khao (White sheath Namwa)	Pale green with pink tinge; whitest sheath overall	Medium (350–380)	11–14 hands per bunch; 14–16 fruits/hand	Angular, long	Raw: Pale green<br>Ripe: Creamy yellow	White flesh, yellow core; sweet and fragrant	Thin-skinned; uniformly large bunches; pale pseudostem color
Kluai Namwa Tanao Sri	Bright green	Very tall (450–480)	14–20 hands per bunch; 14–16 fruits/hand	Large, uniform	Raw: Light green<br>Ripe: Creamy yellow	White fluffy flesh, sweet and aromatic	Resembles Namwa Khao but with larger fruit and bunches
Kluai Namwa Sai Dam	Medium green	Medium (≤320)	5–7 hands per bunch; 12–14 fruits/hand	Long, thick-skinned	Raw: Dull green<br>Ripe: Yellow-orange	Creamy, yellow core; sweet	Contains black sesame-sized seeds in rows
Kluai Namwa Suan (Thong Ma Eng/Thong Loi Ma)	Reddish-brown at base; pink-tinted inner sheath	Medium (300–350)	7–12 hands per bunch	Large, slightly angular	Raw: Dark green<br>Ripe: Bright yellow to golden	White flesh, yellow core; firm, sweet, fragrant	Firm ripe fruits; attractive reddish pseudostem base
Kluai Namwa Tha Yang	-	Medium to very tall (300–500)	15–18 hands per bunch; 22–25 fruits/hand	Large, round-elongated	Raw: Green<br>Ripe: Yellow	Soft flesh, yellow core; sweet and fragrant	Seedless; high fruit count; softer, larger than Mali-Ong
Kluai Nom Mi	-	Short (200–250)	4–7 hands per bunch; 10–16 fruits/hand	Fruit tip irregularly pointed with a large, rounded end	Raw: Green with brown speckled patterns<br>Ripe: Yellow-orange	Dense white flesh; sweet flavor	Thin peel; fruit tip often shows brown splitting; recommended to be cooked before consumption to enhance flavor
Kluai Hak Muk	-	Medium (250–350)	7 hands per bunch; 10–15 fruits/hand	Fruits similar to Kluai Namwa but with sharper, more angular tips	Raw: Thick peel; green<br>Ripe: yellowish-brown (less attractive)	Orange flesh; sweet with slight tanginess	Hybrid traits leaning toward *Musa balbisiana*; thick peel offers good protection but less attractive ripe skin color

Note: The key morphological characteristics of *Musa × paradisiaca* germplasms are based on the studies by Valmayor et al. (2002), Chomchalao and Chattarakom (2004), and Silayoi (2015).

Kluai Nam Wo

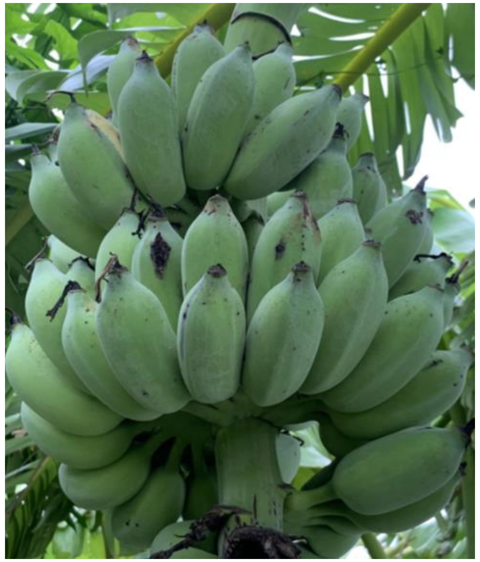

Kluai Hak Muk

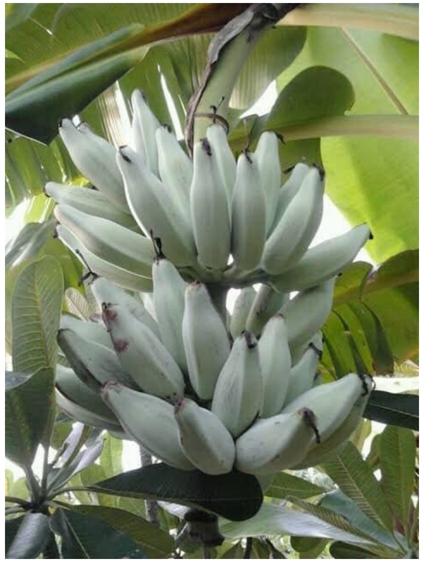

Kluai Nom Mi

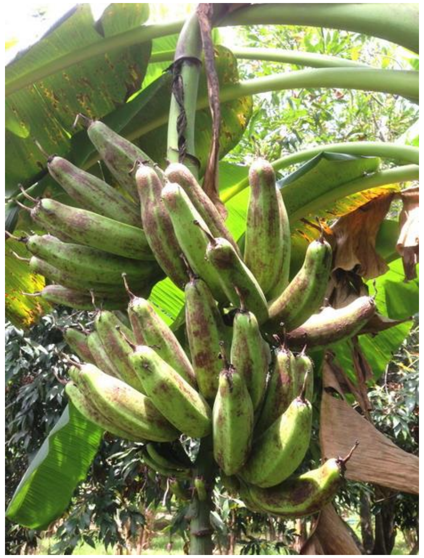

Kluai Hin

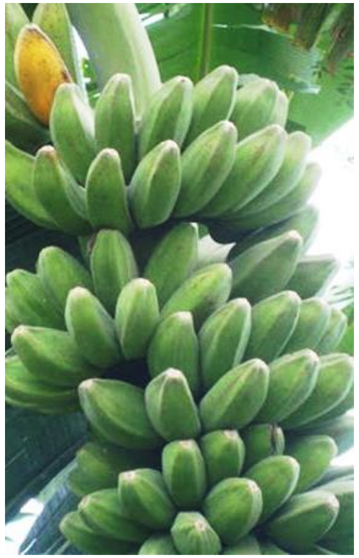

Kluai Namwa Dam

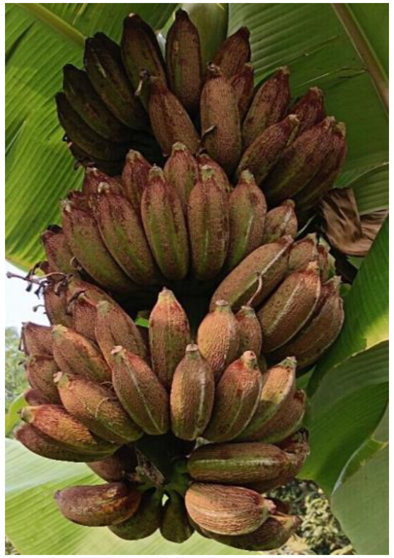

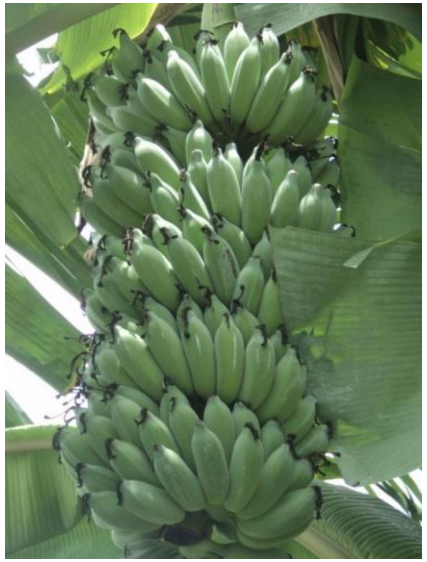

Kluai Namwa Kab Khao

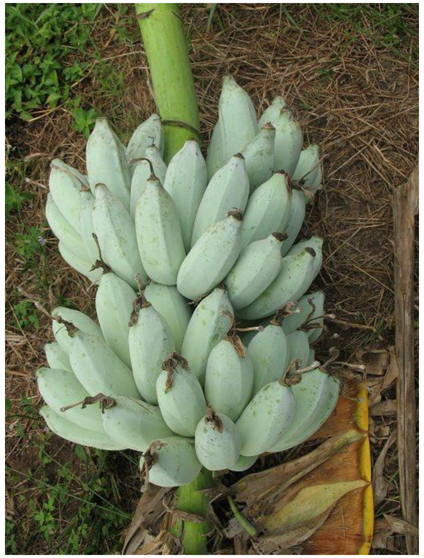

Kluai Namwa Nuan

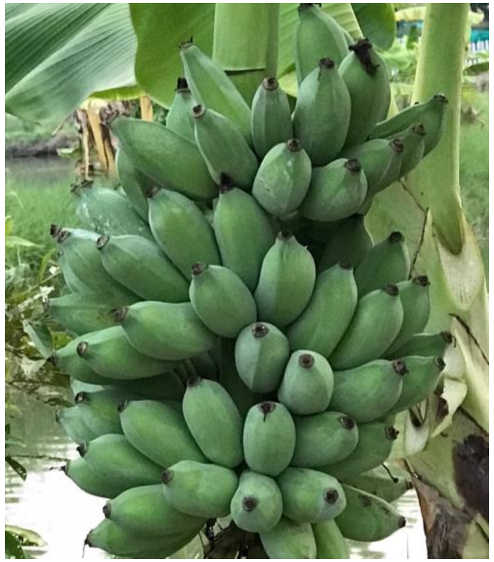

Kluai Namwa Khom

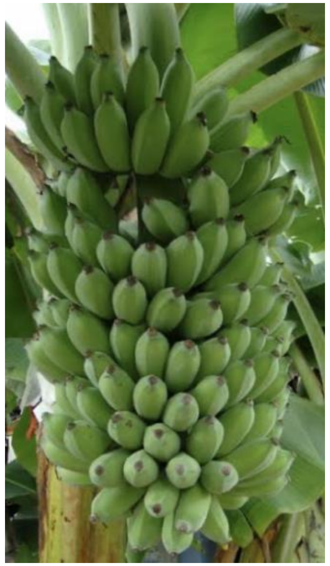

Kluai Namwa Pakchong 50

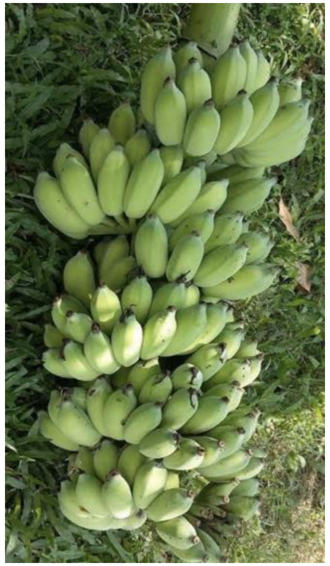

Kluai Namwa Mali-Ong
